# Viral RNA Degradation and Diffusion Act as a Bottleneck for the Influenza A Virus Infection Efficiency

**DOI:** 10.1371/journal.pcbi.1005075

**Published:** 2016-10-25

**Authors:** Max Schelker, Caroline Maria Mair, Fabian Jolmes, Robert-William Welke, Edda Klipp, Andreas Herrmann, Max Flöttmann, Christian Sieben

**Affiliations:** 1 Theoretical Biophysics, Humboldt-Universität zu Berlin, Berlin, Germany; 2 Molecular Biophysics, Humboldt-Universität zu Berlin, Berlin, Germany; 3 IRI Life Sciences, Humboldt-Universität zu Berlin, Berlin, Germany; ETH Zurich, SWITZERLAND

## Abstract

After endocytic uptake, influenza viruses transit early endosomal compartments and eventually reach late endosomes. There, the viral glycoprotein hemagglutinin (HA) triggers fusion between endosomal and viral membrane, a critical step that leads to release of the viral segmented genome destined to reach the cell nucleus. Endosomal maturation is a complex process involving acidification of the endosomal lumen as well as endosome motility along microtubules. While the pH drop is clearly critical for the conformational change and membrane fusion activity of HA, the effect of intracellular transport dynamics on the progress of infection remains largely unclear. In this study, we developed a comprehensive mathematical model accounting for the first steps of influenza virus infection. We calibrated our model with experimental data and challenged its predictions using recombinant viruses with altered pH sensitivity of HA. We identified the time point of virus-endosome fusion and thereby the diffusion distance of the released viral genome to the nucleus as a critical bottleneck for efficient virus infection. Further, we concluded and supported experimentally that the viral RNA is subjected to cytosolic degradation strongly limiting the probability of a successful genome import into the nucleus.

## Author Summary

Influenza A virus carries its segmented genome inside a lipid envelope. Since genome replication occurs inside the nucleus, the main goal of virus infection is to deliver all genome segments through the cytoplasm into the nucleus. After endocytic uptake, influenza viruses transit early endosomal compartments and eventually reach late endosomes. Within a complex maturation process, the endosomal lumen acidifies while the vesicles are transported trough the cytosol. If and how these early processes affect virus infection remained mostly speculative. To reach a better understanding and to quantify the physical interplay between membrane fusion, genome diffusion and infection, we developed a mathematical model that comprises all initial steps of virus infection until genome delivery. We calibrated our model using experimental data and challenged its predictions using recombinant viruses to introduce perturbations. Our results provide a theoretical framework to understand the importance of the endosomal virus passage before membrane fusion and genome release. We further unraveled RNA degradation as a previously unknown limiting factor for virus infection. Our work will help to make predictions and evaluate newly occurring virus strains, regarding their infection efficiency in a given host cell, by simply considering their pH sensitivity.

## Introduction

**Fig 1:**
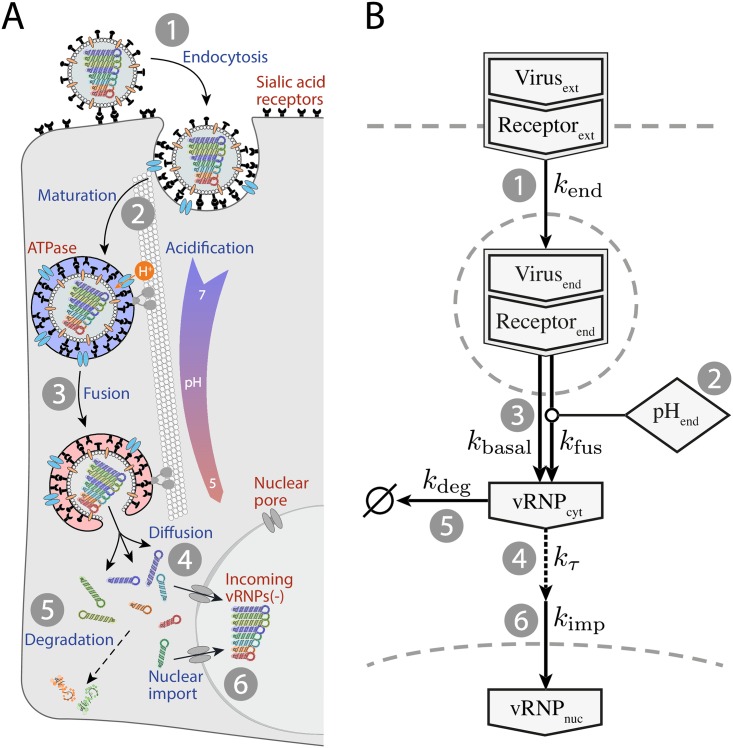
Schematic representation of influenza A virus infection. (A) (1) Influenza virions enter the host cell via endocytosis. (2) During endosomal maturation, the endosomal pH decreases while the endosomes travel along microtubules towards the microtubule organizing center (MTOC), located in the perinuclear region of the cell. (3) When the pH drops below the pH threshold of HA, the protein undergoes a conformational change, triggering fusion between viral and endosomal membrane and the release of the viral genome into the cytosol. (4) The genome, organized in eight vRNPs, needs to diffuse to the nuclear membrane. (5) Diffusing vRNPs are exposed to damage or degradation. (6) The vRNPs that are bound to importin get imported into the nucleus through nuclear pores. (B) Reaction network diagram of the ODE model for virus infection. The corresponding reactions are numbered as shown in panel A.

Seasonal influenza epidemics and periodical pandemics remain a constant threat to the human population. Influenza A virus (IAV) infection is a multi-step process that critically depends on the viral spike protein hemagglutinin (HA), which mediates host cell adhesion by binding to sialic acid-containing receptors within the host cell plasma membrane (Fig 1A). Viruses are subsequently internalized into endosomes, which undergo a complex maturation process involving acidification and centripetal transport along microtubules [1]. Endosomal acidification is highly critical since virus uncoating and genome release depend on membrane fusion mediated by a pH-dependent conformational change of the HA protein. However, although microtubule-associated transport of IAV has been shown before [2–4], the relevance of this directed transport for virus infection still remains largely unclear. The genome of IAV consists of eight individual genome segments coding for a total of 11 viral proteins [5]. The negative-sense single-stranded RNA is packaged together with the viral nucleoprotein (NP) and the polymerase complex (PA, PB1 and PB2) forming rod-like ribonucleoprotein complexes (vRNP). After membrane fusion, released vRNPs travel to the nuclear membrane, most likely by passive diffusion [6]. Since endosomal transport and acidification are concurrent the distance they need to overcome depends on the time and location of membrane fusion and thereby also on the pH-dependent conformational change of HA (i.e. HA’s pH sensitivity). Whether the eight different vRNPs stay in one complex or rather dissociate after being released is still under debate [7, 8]. In any case, the released vRNPs bind to importin-*α* by means of their nuclear localization signals and get shuttled across the nuclear membrane through nuclear pores [7, 9, 10]. To establish a successful infection allowing the production of progeny viruses, it is essential that all eight genome segments are transported into the host nucleus where genome replication takes place. Therefore, the early infection phase may represent a bottleneck for the infection that could potentially contribute to host cell specificity of the virus. Indeed, it was suggested that adaptation to a different host does not only require binding to specific receptors but also modification of the pH sensitivity of HA, possibly to adapt to variations in the endosomal pH [11–13]. Here, we address the question whether an altered pH sensitivity of HA modulates the residence time of diffusing vRNPs in the cytoplasm and thereby influences viral infectivity.

Mathematical models have been used before to foster a better understanding of IAV replication on the population [14, 15] as well as on the single-cell level [16–19]. However, none of those seem to be adequate for the analysis and comparison of different IAV strains with varying pH sensitivity.

We developed a model accounting for the first critical phase of IAV infection using a combination of ordinary differential equations (ODE) and a spatial modeling approach. The model parameters were calibrated using experimental data from infection studies covering the individual steps of the infection process (Fig 1A). Our model enabled us to (1) predict differences in the infection efficiency (i.e. delivered number of vRNPs in the nucleus) of two IAV strains with altered pH sensitivity and to (2) simulate the diffusion of vRNPs as complex or individual particles utilizing a spatial stochastic model accounting for the specific cell geometry as well as the stochastic nature of the underlying processes.

Our simulations predicted that the pH sensitivity of HA critically controls the time of fusion and thereby the location of genome release. We further concluded that a dissociation of released vRNP segments is highly unlikely to result in an efficient infection and that the viral RNA (vRNA) is subjected to degradation during cytosolic transport. This in turn might affect the number of intact vRNPs arriving in the nucleus and thus the infection efficiency of a specific virus. Using two HA variants with differing pH sensitivities, we complemented our simulations with experimental evidence validating the major model predictions.

Taken together, we propose that the pH dependence of influenza virus fusion resulting in the release of the viral RNP complex plays a determinative role for the initial phase of virus infection. Our modeling data further suggest that diffusive transport and cytosolic stability of vRNPs represent limiting factors for efficient infection.

## Results

### A dynamic model reproduces IAV entry dynamics

To understand how a varying pH sensitivity can affect infection efficiency, we need to understand the dynamics of the entry process after virus binding. To this end, we developed a dynamic ODE model that accounts for the early steps of the infection cycle and parameterized it using experimental time course data. The model consists of a set of four coupled ODEs describing (1) the temporal behavior of the amount of surface bound virions, (2) the quantity of endocytosed viruses, and (3) the concentration of released vRNPs in the cytoplasm as well as (4) in the nucleus. It describes the processes of endocytosis, endosome maturation, endosomal fusion and nuclear import of vRNPs. Our experimental data led us to include cytosolic vRNP diffusion and degradation into the model. The reaction network is depicted in Fig 1B, and the corresponding equations are introduced in the Materials and Methods section.

**Fig 2:**
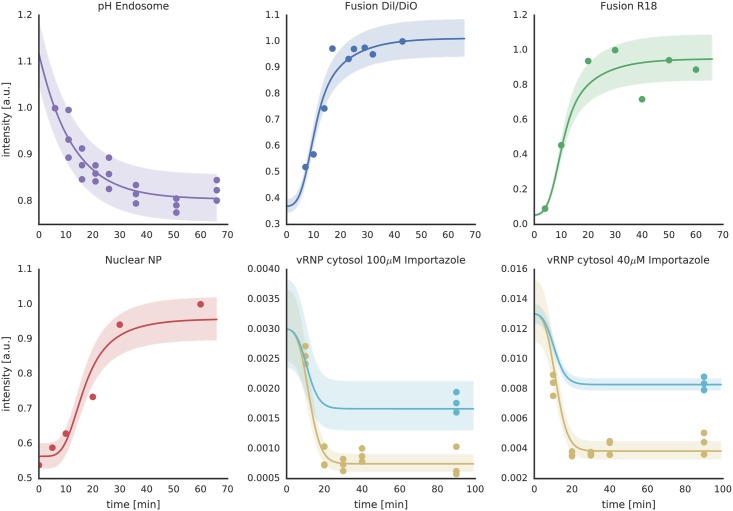
Experimental data (symbols) and resulting ODE model fits (solid lines) for the IAV infection model. Shown are time course data for endosomal pH, IAV-endosome fusion as measured by DiO/DiI and R18 dequenching as well as nuclear NP accumulation after virus uncoating. Furthermore, the cytosolic vRNA lifetime was assessed by quantitative RT-PCR after infection while blocking nuclear import using importazole (blue, total vRNA level; brown, cytosolic vRNA level). The shaded areas represent the estimated experimental error based on a parametric error model (see Materials and Methods). All y-axes show signal intensities of the respective measurement readout in arbitrary units (a.u.). For the underlying model trajectories refer to S5 Fig.

To calibrate our model, we measured the corresponding steps of virus infection—virus-endosome fusion, endosomal pH development, and nuclear NP accumulation—between 0 and 40 minutes post infection (p.i.) (Fig 2). Intracellular fusion was measured by infecting MDCK cells using viruses labeled with R18 at self-quenched concentration. Individual viruses were detected using confocal microscopy and fusion events were counted using automated image processing. R18 dequenching is a commonly used method to detect IAV fusion *in vitro* [20] and a significant increase in R18 intensity was expected upon virus-endosome fusion (S1 Fig). Indeed, we observed that the intensity of internalized single viruses showed a strong increase between 10 and 20 min p.i. reaching a plateau after about 25 min (S2 Fig). Notably, we could reproduce the virus-endosome fusion kinetics using a FRET-based fluorescence readout as described previously [21]. This trend correlated with the accumulation of viral NP inside the nucleus, which started to increase slightly delayed after 15–20 min (Fig 2) as detected by anti-NP immunostaining.

In order to identify pH-dependent effects on IAV infection, it was crucial to know the actual endosomal pH dynamics. In the model, we defined the endosomal pH as an independent variable pH_end_, which served as an input to the coupled reaction system. pH_end_ followed an exponential decay, that we fitted to experimental data, with a decay parameter *k*_ATPase_ that corresponds to the endosomal V-ATPase activity.

To monitor the endosomal pH kinetics and calibrate *k*_ATPase_, we used a double-labeled dextran as a pH-sensitive endosomal probe as described in Materials and Methods. Briefly, after a short starvation period, cells were loaded with dextran for the indicated time points, washed and immediately analyzed by flow cytometry. We observed an exponential decrease of the endosomal pH over the observed time period (Fig 2). HA’s membrane fusion activity is characterized by a narrow pH regime of switching from zero to complete fusion [22]. To accommodate for this behavior, in our model, endosomal fusion follows a Hill kinetics [23] with the threshold parameter *k*_H+_ and the Hill coefficient *h*. We could determine these parameters by measuring the viral fusion kinetics *in vitro* using R18 fluorescence dequenching (FDQ) in the pH range from 5.0 to 7.4. We observed a steep increase of viral fusion with decreasing pH (S3 Fig) and could use our model to determine the fusion pH threshold value *k*_pH_ (i.e. the pH value where FDQ is half-maximal) of the IAV X-31 strain to be at pH 5.6 from these data sets.

The combination of results from *in vitro* virus fusion with the endosomal fusion time course data allowed us to fit the parameters of the model with high confidence (see Table 1 and S4 Fig) further enabling us to introduce perturbations into the model. During model development, it became evident that introduction of vRNP diffusion delay and degradation is necessary to fit all measured data at once.

**Table 1:** Parameter names, optimal values, upper and lower bounds of 95%-confidence intervals and units are given for all kinetic model parameters.

Parameter	$\hat{\theta }$	conf_lb_	conf_ub_	Unit
*k*_ATPase_	7.94 × 10^−2^	5.05 × 10^−2^	1.17 × 10^−1^	[min^−1^]
*h*	1.90	1.39	2.62	[1]
*h*_WSN_H3_mut_	4.25	3.02	6.67	[1]
*h*_WSN_H3_wt_	2.29	1.72	3.15	[1]
*k*_H+_	4.86 × 10^−6^	3.38 × 10^−6^	1.01 × 10^−5^	[mol⋅l^−1^]
*k*_H+_WSN_H3_mut_	2.10 × 10^−6^	1.79 × 10^−6^	2.91 × 10^−6^	[mol⋅l^−1^]
*k*_H+_WSN_H3_wt_	4.34 × 10^−6^	3.34 × 10^−6^	8.11 × 10^−6^	[mol⋅l^−1^]
*k*_basal_	9.24 × 10^−3^	5.66 × 10^−3^	1.31 × 10^−2^	[min^−1^]
*k*_deg_	1.89 × 10^−1^	5.68 × 10^−2^	∞	[min^−1^]
*k*_end_	9.32 × 10^−2^	4.28 × 10^−2^	∞	[min^−1^]
*k*_fus_	3.59 × 10^−1^	2.79 × 10^−1^	7.11 × 10^−1^	[min^−1^]
*k*_imp_	1.00 × 10^4^	0.00	∞	[min^−1^]
*k*_inhib_100*μ*M_	1.84 × 10^−4^	0.00	∞	[1]
*k*_inhib_40*μ*M_	9.89 × 10^3^	0.00	∞	[1]
*k*_*τ*_	1.21	5.04 × 10^−1^	∞	[min^−1^]
pH_lb_	4.46	4.01	4.80	[log_10_(mol ⋅ l^−1^)]
pH_ub_	6.20	5.65	6.91	[log_10_(mol ⋅ l^−1^)]

The confidence intervals were determined using the profile likelihood approach [44]. The plots of the likelihood profiles are depicted in S4 Fig. For additional parameters and parameter ranges refer to S1 Table.

### The pH sensitivity of HA affects viral infection efficiency in MDCK cells

In order to predict the system’s behavior for a higher value of *k*_pH_ resulting in earlier virus-endosome fusion, we initially did not include vRNP diffusion and degradation into the model. In that case an unchanged steady state of accumulated vRNPs in the nucleus vRNP_nuc_ was predicted. Hence, the pH sensitivity had no measurable effect on virus infection in that model. To experimentally determine the effect of an altered pH sensitivity of HA on viral infectivity, we constructed a recombinant virus based on influenza virus A/WSN/33 (H1N1) with the H1-subtype HA replaced by H3 HA of A/X-31 (H3N2) yielding the so-called WSN H3 wild type virus (WSN H3 wt) [24] as well as a WSN H3 mutant virus carrying the destabilizing double mutation T212E-N216R in the HA protein (WSN H3 mut) [25]. The fusion pH threshold of WSN wild type and mutant viruses was assessed *in vitro* as described above for A/X-31.

**Fig 3:**
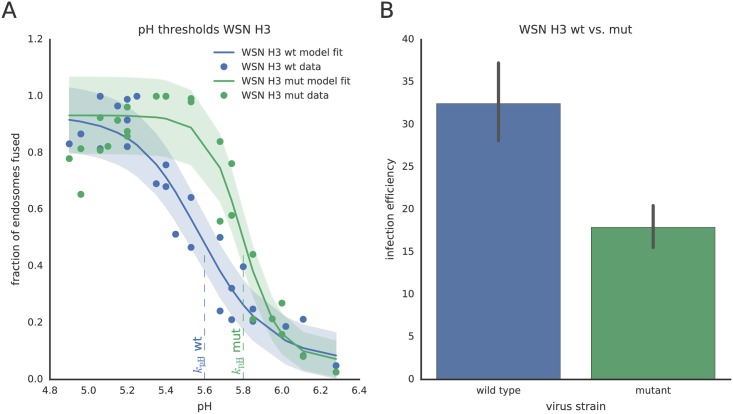
Virus-cell fusion and infection efficiency of WSN H3 wild type and mutant viruses. (A) *In-vitro* pH-dependent virus-cell fusion efficiency of recombinant WSN H3 wt and WSN H3 mut viruses (H3 T212E-N216R). Symbols denote measured data, solid lines show the results of the ODE model fitting. The shaded areas indicate one standard deviation of the measurement noise of the respective measurement technique. The pH thresholds of fusion, i.e. the pH at half-maximal fusion efficiency are indicated by the dashed lines at 5.6 (wt) and 5.8 (mut). (B) Comparison of the infection efficiency of WSN H3 wt and mut in MDCK cells as measured by viral NP accumulation upon infection. MDCK cells were infected at MOI 0.2. 20h post-infection, cells were fixed, stained using anti-NP antibodies and counted after fluorescence microscopy. Data in B show mean and SEM from four independent experiments.

Our experiments showed that, similar to the A/X-31 strain, a pH threshold of 5.6 triggers fusion of the WSN H3 wild type strain confirming that fusion mainly depends on the pH sensitivity of HA (S3 Fig). In contrast, for the WSN H3 mutant, this threshold was shifted to pH 5.8 due to the destabilizing double mutation in the HA protein (Fig 3). We integrated these data into the dynamic model and again could not detect an effect on nuclear vRNP accumulation suggesting—at first glance—no effect of HA’s pH sensitivity on virus infection.

However, from measuring the infection efficiency of recombinant WSN viruses in MDCK cells under identical conditions (MOI of 0.2), we found that the amount of expressed viral NP 20h p.i. was 40–50% lower for the destabilized WSN H3 mutant virus than for the wild type (Fig 3B). Both, measuring the infection efficiency 5h p.i. as well as growth curves for both virus strains over 72h, could confirm the attenuation of WSN H3 mut (S6 Fig). If we considered a correlation between accumulated vRNPs in the nucleus and expressed NP protein 20h p.i., these results clearly contradicted our model prediction. Therefore, we extended our model to reproduce the infection efficiency observed in the experiment. Based on our experimental observations of virus-endosome fusion taking place some *μ*m away from the nucleus (S7 Fig), we introduced a spatial component that accounts for the diffusion of released vRNPs through the cytosol.

### A spatial model yields insights into the vRNP diffusion process

We constructed a 3D reaction-diffusion model that allowed us to relate fusion distance to delay and efficiency of vRNP delivery into nucleus and to compare the different IAV strains quantitatively. The simulation settings were adjusted to biologically relevant values (see Materials and Methods section). For the assumed cell geometry as well as the position of the nucleus, please see S8 and S9 Figs. Due to the small number of vRNP particles (1 to about 80) in the cell and a binary outcome (infection/no infection) for each cell, we could not assume a mean-field approach to capture the full behavior of the system and thus decided to utilize a stochastic simulation environment. To explain the experimental result of reduced infection efficiency, we established the hypothesis that free vRNPs are subjected to degradation while diffusing through the cytosol [26]. We further allowed two forms of vRNP diffusion: (1) vRNPs are released from the virus as a complex consisting of all eight genome segments diffusing together. (2) The released complex dissociates and the vRNP segments diffuse individually with a higher diffusion coefficient than the larger complexes. The infection is only counted as successful if a full set of eight different vRNPs has reached the nuclear membrane where they bind to importins to be shuttled into the nucleus.

**Fig 4:**
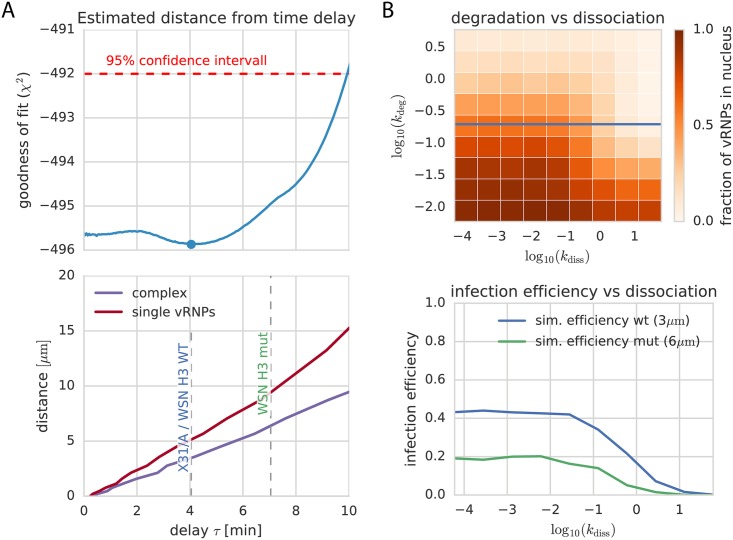
Combination of ODE and diffusion model. (A, upper panel) The profile likelihood of the delay time between membrane fusion and vRNPs reaching the nucleus (*τ*) as resulting from our ODE model (see profile likelihood for *k*_*τ*_ in S4 Fig) shows a minimum at 4 min. We can use this value to calculate a mean fusion distance using our 3D diffusion simulations (A, lower panel). We obtain an estimated distance of 5 *μ*m for dissociated single vRNPs and 3 *μ*m for the full complex. (B) Scanning the parameter space of the spatial model around the values suggested by the ODE fits shows a strong dependence of dissociation (*k*_diss_ = [10^−4^, 10^−1^]) and degradation (*k*_deg_ = [10^−6^, 1]) during diffusion. Both of which would lead to strongly inhibited infection efficiency. The blue line shows the experimentally identified degradation rate. To better compare the infection efficiency of both used virus strains, the lower panel shows simulations for different dissociation constants with the predicted distances for WSN H3 wt and mut. The estimated distance difference leads to a 50% lower infection efficiency in our simulations.

In our spatial model, three parameters were critical for the connection between infection efficiency and pH sensitivity: (1) vRNP degradation, (2) vRNP release distance from the nucleus and (3) vRNP dissociation. Our computational approach made it possible to comprehensively sample a large sub-space of combinations of different dissociation rates of vRNP complexes (*k*_diss_) and degradation rates of individual or complexed vRNPs (*k*_deg_). Fig 4B shows the results of such two-dimensional parameter scans. The *x*- and *y*-axes indicate the parameter values of *k*_diss_ and *k*_deg_. Each color-coded bin corresponds to the average number of successful infections after 40 min in a cell population of 1000 cells. The populations were all simulated with the corresponding parameters on the axes. The initial condition for the simulation is one complete vRNP package starting to diffuse at distance *d*_nuc_ from the nucleus (S10 Fig). Interestingly, these parameter scans suggested that, given the measured diffusion rates, already small degradation rates would be sufficient to degrade the whole viral genome if virus-endosome fusion and thus vRNP release happens too far away from the nucleus.

Furthermore, although single particles have a much higher mean displacement due to their smaller size, our simulations clearly showed that the dissociation of complexes lowered the probability of a complete genome in the nucleus. The larger the dissociation constant in the model was set, the lower the percentage of complete genomes. This effect also increased with larger fusion distances from the nucleus, possibly because of an increased probability that the vRNPs were degraded before reaching the nucleus.

### Cytosolic vRNA degradation can lead to reduced infection efficiency

Our observations at the cell population level (Fig 3B) revealed that the infection efficiency of an IAV strain with a higher pH threshold was reduced as compared to the reference strain (WSN H3 wt). By adding a degradation term to the freely diffusing vRNP particles, we could establish a link between the pH sensitivity of HA and the viral infection efficiency: the larger the distance between the location of fusion and the nucleus, the higher the probability that the vRNPs are degraded and thus the lower the number of functional vRNPs arriving in the host cell’s nucleus.

To test whether vRNPs are subjected to degradation while diffusing in the cytoplasm, we measured the amount of cytosolic vRNA (HA segment) by RT-qPCR while blocking the nuclear import using 40 and 100 *μ*M importazole, respectively [27, 28] (for experimental details see Materials and Methods). The resulting data are shown in Fig 2. As also reported by Kublun *et al*. [28], we could observe a higher inhibitory effect on nuclear import using 40 *μ*M importazole than using the higher concentration of 100 *μ*M which might be due to cell damage or the activation of other import pathways for the higher dose of the drug. In any case, for both importazole concentrations the results indicate a fast drop of cytosolic (including intact viral particles) vRNA in the first 10 min p.i.. We also compared the total (nuclear + cytosolic) vRNP concentration with and without importazole 60 min p.i. showing that with importazole the vRNP levels are significantly lower (for control experiments without importazole see S11 Fig).

By including these data into the fit of our ODE model, we could identify the model parameters accounting for vRNA degradation (*k*_deg_) and diffusion time (*k*_*τ*_) as well as their confidence intervals (S4 Fig, Table 1). With these parameters we could use the spatial model to further characterize the impact of fusion distance on infection.

### Combination of ODE and spatial model can predict limits for successful infection

Combining the results of the ODE and the 3D diffusion model enabled us to determine the mean fusion distance of vRNP particles in our simulations. Based on the calibration data for the ODE model, we could estimate an optimal delay time between membrane fusion and vRNP arrival at the nucleus *τ* of 4 minutes for IAV X-31 (Fig 4A, upper panel). Using the spatial model simulations, we could then translate this value into a distance of fusion from the nucleus of ≈ 3 *μ*m for the complex and ≈ 5 *μ*m for individually diffusing vRNPs (Fig 4A, lower panel).

**Fig 5:**
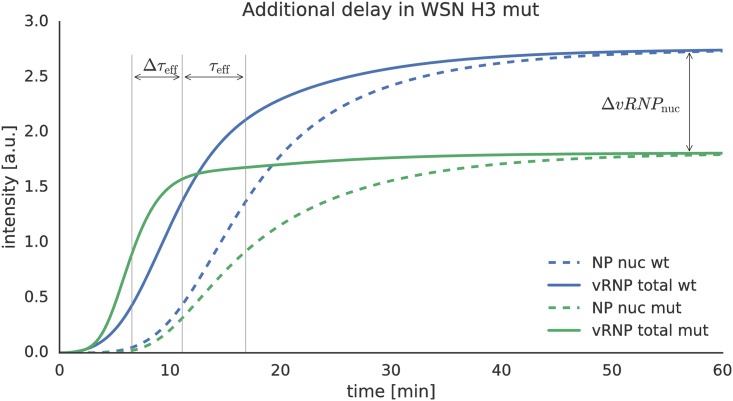
Effect of different HA pH sensitivity values on the vRNP diffusion time and number of arriving vRNPs in the nucleus. The fitted value for *τ* (Fig 4) is depicted as the time difference between fusion (solid lines) and nuclear import (dashed lines). The green curves show the simulated dynamics for the WSN H3 mutant virus. The estimated Δ*τ*_eff_ for WSN H3 mut is around 3 min, which would lead to significantly lower nuclear vRNP levels due to their degradation in the cytosol. The y-axis shows the signal intensity of the respective simulated parameter.

In the simulations, the estimated diffusion delay parameter *k*_*τ*_ for IAV X-31 represents the delay between the half maximum times of fusion and import into the nucleus which we denote as *τ*_eff_ (Fig 5). It is reasonable to assume that the vRNPs of WSN H3 wt and H3 mut have the same diffusion coefficient and import rate (*k*_imp_) as the one fitted for IAV X-31. Furthermore, for the higher pH threshold of WSN H3 mut, an increase of the vRNP concentration in the nucleus can at best happen as early as for the wild type because we assume active transport to be faster than diffusion. Using our model, we can now combine endosome acidification data and HA pH sensitivity measurements to predict the dynamics of the mutant infection. Simulations showed the mutant fusing about three minutes earlier than the WSN H3 wt (Δ*τ*_eff_ ≈ 3 min) which corresponds to a fusion distance of ≈ 6 *μm* for the complex. If we simulated the model for the mutant with a prolonged delay *τ*_mut_ ≈ *τ*_wt_ + Δ*τ*_eff_ we could predict a strong reduction in the amount of vRNPs reaching the nucleus, which could be a possible explanation for the reduced infection efficiency observed for the mutant.

Next, we used the estimated degradation rate (*k*_deg_ = 0.19 min^−1^) in the stochastic model to estimate upper limits for fusion distances from the nucleus for a successful virus infection by simulating the diffusion of cytosolic complexed vRNPs from different distances. We found that for a fusion distance greater than *d*_nuc_ = 10 *μ*m from the nucleus, on average only 0.4 complete sets of viral genomes would arrive in the nucleus when running the simulation with one complete set. The number of complete sets quickly reached zero with larger distances. Simultaneously, we could scan through the parameter *k*_diss_ to obtain a parameter landscape of successful infection (Fig 4B). Again, the dissociation of vRNPs affected transport to and import into the nucleus and proved to be detrimental for successful infection: for *k*_diss_ > 0.1 nearly no vRNPs would reach the nucleus even for small distances.

## Discussion

Viral escape from cellular endosomes is an essential step of the IAV replication cycle. To allow uncoating and vRNP release, IAV utilizes the complex maturation program of the endocytic system. Among other changes, it involves two inherently connected processes: endosomal motility and lumenal acidification [1].

Endosomal motility is dominated by the association with and movement along microtubules. This transport is mediated by two motor protein families, kinesins and dyneins, which operate in opposite directions. Although endosomes typically associate with both motors, which can lead to a bidirectional movement, they perform a net translocation towards the microtubule organizing center (MTOC) in the nuclear periphery [1]. Indeed, live-cell tracking of fluorescently labeled IAV showed that the particles are transported towards the perinuclear region of the cell [2, 4].

During the directed transport inside endosomal vesicles, the virus finally reaches late endosomal compartments (pH 5.0-6.0) where the acid-induced conformational change of HA triggers membrane fusion and vRNP release into the cytosol. Due to the connection between lumenal acidification and endosome movement towards the nucleus, timing of fusion affects the location of the fusion event inside the cell.

Several studies suggested that the pH sensitivity of HA needs to be adapted to the endosomal properties of the respective host cell in order to allow for efficient virus replication [11–13]. However, so far it remained largely unclear how HA’s pH sensitivity affects time and location of fusion and as a consequence, the infection efficiency of the virus.

Here, we present a mathematical model that quantitatively describes IAV cell entry. We combined cell-specific parameters like geometry, endosomal uptake and acidification with virus-specific determinants such as the pH sensitivity of HA and were able to estimate the distance of endosomal fusion from the nucleus for different HA variants. Furthermore, we showed that vRNA is subjected to cytosolic degradation and thereby demonstrated the importance of the exact timing of fusion for the efficiency of infection in a given host cell.

Measuring the intracellular fusion kinetics of IAV X-31, we found a mean fusion time of 10 min in MDCK cells which is in good agreement with Lakadamyali *et al*. [2] who reported a mean fusion time of 8 min in CHO cells. Our result also correlates well with the study of Sakai *et al*. [21], who used the same intracellular fusion assay, although in a different cell line. As reported previously [2, 4], fusion events were detected close to the nucleus, which we could demonstrate to be on average located in the center of the cell (S7 and S8 Figs).

Using our combined stochastic and spatial model, we could predict that IAV X-31 and a recombinant WSN virus carrying the H3 HA of IAV X-31 fuse at an estimated distance of 3 *μ*m from the nucleus in MDCK cells, whereas a more pH sensitive mutant (*k*_pH_ = 5.8 compared to 5.6 for the wild type) fuses earlier at a distance of 6 *μ*m from the nucleus. We proposed that the released vRNPs are subjected to degradation, thereby strongly attenuating the infection as well as the replication efficiency of the WSN H3 mutant virus as observed experimentally (Fig 3 and S6 Fig). Indeed, we could show that vRNAs, complexed in diffusing vRNPs after being released from the virus, are degraded within a few minutes in the cytosol (degradation rate of 0.19 min^−1^). Adding this limiting factor to our model allowed us to predict that the earlier release of the viral genome, i.e. in larger distance to the nucleus (due to a higher pH sensitivity of HA), results in a lower level of nuclear vRNPs and thus, in a lower infection efficiency.

It was shown previously that IAV propagation over multiple passages can lead to accumulation of defective interfering RNAs, which give rise to defective interfering particles (DIP) that interfere with the replication of non-defective viruses [29, 30]. Since the presence of DIPs and in particular deletions in HA and NP could affect our results, we performed a segment-specific PCR. We included the PB2 segment since DI RNAs predominantly originate from the large polymerase genes (segments 1–3) [31]. In addition to all three full length segments, for PB2 we found a smaller PCR product indicating the presence of DIPs in our virus sample (S12 Fig). However, for both the HA as well as the NP segment, we could not detect a significant amplification of smaller PCR products, thereby ensuring the unperturbed detection of HA vRNA as well as incoming NP protein during our virus-entry measurements.

With our model, we were also able to challenge existing hypotheses on the different strategies of how vRNP segments reach the nucleus (i.e. as a complex or as individual vRNPs). Hence, we simulated various degrees of dissociation of vRNP segments after fusion and compared the effect on infection efficiency. Our model suggests that diffusion of complexed vRNPs is more favorable for infection than dissociation of vRNPs in the cytosol. Transport as a complex might assure the arrival of all eight vRNPs in the nucleus simultaneously and thus be much more efficient and reliable than gating single vRNPs one-by-one. Our model thus supports the tight association of released vRNP segments during cytosolic diffusion as it was also recently suggested from single-molecule fluorescence in situ hybridization (smFISH) data [8].

Our mathematical model integrates cell-specific as well as virus-specific parameters. Therefore, it is useful to predict the infection of a given virus in a specific host cell. Mathematical models of virus entry have been presented for Semliki Forest virus in BHK-21 cells and of the baculovirus-insect cell system [32, 33]. These viruses—similar to influenza—require endocytic uptake and, after release of the viral genome by membrane merger, transport to a specific compartment for replication. Our model extends the current understanding of the role of the fusion protein’s pH sensitivity on viral entry dynamics and the impact of vRNA degradation as well as vRNP dissociation for the success of infection. These newly determined parameters might also be relevant for other models of viral entry. Still, the complex nature of a given host cell and the possible presence of other yet unknown factors of the cellular immune response may lead to deviations from our model predictions. For example, the activation of signaling pathways upon viral binding can interfere with the uptake of the virus what would considerably influence the dynamics of viral entry. The impact of such factors on infection can vary greatly between different viruses and cell lines and thus is difficult to predict. Furthermore, in this study we only focused on the early steps of IAV infection, which limits its predictive power for viral replication. To improve predictions for this case the model could be combined with existing models that describe the whole infection cycle [34], which might be very useful for several applications e.g. vaccine production [16].

Taken together, we describe the first critical phase of IAV infection using mathematical and 3D diffusion modeling, which accounts for the pH sensitivity of HA as well as cell-specific parameters such as endosomal acidification, cell geometry and the degradation rate of vRNPs, a newly identified host factor of the cellular immune system. Using recombinant viruses with differing HA pH sensitivities we could validate our model experimentally and at the same time provide evidence that the interplay between pH-dependent fusion of HA and degradation of viral RNA by the cellular immune system represents a bottleneck for IAV entry determining the success of viral infection.

## Materials and Methods

### Experimental methods

#### pHW2000 plasmids for generation of recombinant viruses

pHW2000 plasmids containing the viral proteins of A/WSN/33 (H1N1) were kindly provided by Dr. Michael Veit (Free University Berlin). To obtain pHW2000-plasmids containing the H3 HA segment of IAV X-31, the viral RNA was extracted from infected cells using the QIAGEN RNeasy Kit. Viral cDNA was subsequently synthesized by reverse transcription using the Uni12 primer [35]. The cDNA of H3 HA was amplified using segment-specific primers and cloned into the pHW2000-plasmid using the BsaI restriction enzymes as described previously [36]. The T212E-N216R mutation was inserted according to the QuikChange mutagenesis protocol from Stratagene. Correct insertion of the H3 HA segment into pHW2000 and presence of the double-mutation was confirmed by sequencing (GATC Biotech AG, Germany).

#### Material, cell and virus culture

Madin-Darby Canine Kidney (MDCK) cells and HEK-293T cells were cultured in Dulbeccos Modified Eagles Medium (DMEM) without phenol red, supplemented with 1% penicillin/streptomycin and 10% fetal calf serum (FCS). The cells were passaged every 3–4 days. One day prior to the experiment, the cells were detached from the cell culture flask using 0.5% Trypsin/EDTA for about 10 min. The cells were diluted in DMEM and 2 − 5 × 10^5^ cells were seeded in 35mm poly-L-lysine coated glass bottom petri dishes (MatTek Corp.). Influenza A (H3N2) X-31 was propagated in chicken eggs (TCID50 7.5 × 10^6^)and recombinant A/WSN/33 containing wild type or mutant H3 HA (WSN H3 wt/mut) in MDCK cells. Prior to the experiment the virus was diluted to 1mg/ml protein concentration. Octadecylrhodamine B (R18) was purchased from Molecular Probes (Life Technologies, USA). Phosphate buffered saline (PBS) was used for all dilutions during the experiments. FITC/Tetramethylrhodamine (FITC/Rho) and Tetramethylrhodamine dextran were purchased from Life Technologies (USA). Human erythrocytes where purchased from Blutbank Charité Berlin. For assessment of the HA titer, erythrocytes were washed, diluted to 1% in PBS and incubated with a series of two-fold dilutions of an IAV sample in a 96-well plate format.

#### Recombinant viruses

Recombinant A/WSN/33 viruses were produced by using the eight-plasmid system as described previously [24]. The plasmid encoding for the HA protein of A/WSN/33 (H1 HA) was exchanged for the newly constructed pHW2000- H3 wild type (wt) or -H3 T212E-N216R (mut) plasmids to obtain WSN H3 wt or mut viruses, respectively. Briefly, HEK-293T cells were transfected with 0.5 *μ*g of each of the eight plasmids in a 35mm dish. After 6h of incubation at 37°C and 5% CO2 the medium was exchanged to virus cultivation medium (DMEM with 0.1% FBS, 0.2% BSA, 2mM glutamine, 1% penicillin/streptomycin and 1 *μ*g/ml TPCK Trypsin). After 2 days the virus was harvested and amplified in MDCK cells. Viral titers were determined using HA and TCID50 assay (S6 Fig). The infection efficiency of recombinant viruses was determined by immunostaining against the viral nucleoprotein 20h post-infection as described below.

#### Intracellular fusion assay

Influenza virus was diluted to a final protein concentration of 1mg/ml in PBS and incubated with 20 *μ*M R18 (Sigma) or DiI/DiO (Life Technologies) at a ratio of 2:1 (66 *μ*M/33 *μ*M) for 30 min at room temperature. Unbound dye was removed by centrifugation at 25.000 g for 5 min or gel filtration (G25 Sephadex in PBS). The virus was resuspended in PBS and stored at 4°C. MDCK cells were washed with PBS and incubated with infection medium (serum-free DMEM, 0.2% BSA) for 30 min, then washed and placed on ice. Immediately before the experiment, the virus was diluted to 40 *μ*g/ml in infection medium and viral aggregates were removed using a 0.2 *μ*m sterile filter. The labeled virus was applied to the cells, allowed to bind for 10 min before the temperature was raised to 37°C. The described cell treatment does not interfere with the morphology of the cytosceleton as well as bulk endocytosis and vesicle transport (S13 and S14 Figs). The cells were imaged at 37°C using a confocal microscope. Acquired were at least 5 fields on view (10–20 cells each) as z-stacks per time point. The boundaries between cells were determined from the bright field image. The signal from individual viruses in projected z-stacks was analyzed using an IDL-based particle identification software [37] and ImageJ.

#### Immunostaining

For the determination of the nuclear NP accumulation, cells were infected with unlabeled virus as described in the previous paragraph on intracellular fusion. MDCK were washed in PBS buffer and fixed in PBS containing 2% paraformaldehyde and 0.2% glutaraldehyde for 20 min. The cells were permeabilized with PBS containing 0.2% Triton X-100 and 0.2% BSA for 20 min, washed in PBS and incubated with anti-Nucleoprotein (Millipore, USA) antibody for 1 hour. The cells were washed in PBS and incubated with the secondary anti-mouse Cy2 or Alexa488 conjugate antibody for 1 hour (Amersham, GE, USA). Finally, the cells were counterstained using PBS containing 0.2 *μ*g/ml DAPI for 10 min.

#### Influenza virus-cell fusion assay

Virus-cell fusion was measured by monitoring the fluorescence dequenching (FDQ) of the lipid-like fluorophore R18 upon fusion of R18-labeled viruses with ghost membranes [22]. To this end, 10 *μ*l of labeled virus suspension (1mg/ml) were mixed with 40 *μ*l ghost suspension (≈ 2 × 10^5^ cells) and incubated for 20 min at RT. Unbound virus was removed by centrifugation (5 min, 2000g). The virus-ghost suspension was transferred to a glass cuvette containing pre-warmed fusion buffer (pH 7.4), and the fluorescence was detected (*λ*_*ex*_ = 560nm; *λ*_*em*_ = 590nm) by using a Horiba Yobin Yvon FluoroMax spectrofluorometer. Fusion was triggered by the addition of citric acid (0.25M). The suspension was stirred continuously with a 2 by 8mm Teflon-coated magnetic stirring bar. After 600s the fusion reaction was stopped by adding Triton X-100 (50 *μ*l, final concentration 0.5%) to obtain maximal R18 dequenching. The final pH in the cuvette was measured using a standard pH meter. The fraction of FDQ was calculated as:

$$\text{FDQ}=\frac{F(t)-F(0)}{F_{\text{max}}-F\left(0\right)}$$ (1)

where *F*(0) and *F*(*t*) are the fluorescence intensities before fusion and at a given time *t*, respectively (Fig 3A and S3 Fig).

#### Endosomal pH determination

One day prior to the experiment, MDCK cells were seeded into 35 mm poly-L-lysine coated glass bottom petri dishes (MatTek Corp.). For dextran labeling, the cells were washed with PBS and incubated in serum-free medium for 30 min at 37°C, followed by 5 min with 10mg/ml double-labeled dextran at 37°C (pulse). We tested and found that the cell starvation does not perturb bulk endocytosis and vesicle transport (S14 Fig). After the pulse, the cells were washed in cold PBS and resuspended in pre-warmed cell culture medium. After the indicated time points, cells were detached, diluted in cold PBS and analyzed by flow cytometry.

#### Fluorescence microscopy

For fluorescence microscopy, we used an Olympus FV1000-MPE confocal microscope (Olympus, Japan) equipped with 405nm (DAPI), 440nm (CFP), 488nm (GFP), 559nm (R18) and 635nm (A647) laser lines, an Olympus 60x/1.2 water UPlanSApo objective and 405-458/515/559/635 405/488/559/635 dichroic mirror filter sets.

#### Isolation of cytosolic RNA from importazole-treated cells

MDCK cell were treated with 40 *μ*M [27] or 100 *μ*M importazole [28] (Sigma, Taufkirchen Germany) for 30 min at 37°C and 5% CO2 followed by an incubation with IAV X-31 for 10 min on ice. After viral adhesion the supernatant was removed and cells were washed with PBS. Cells were subsequently incubated with 1U/ml Neuraminidase (Sigma, Taufkirchen Germany) for 10 min at 37°C and 5% CO2 to remove viruses that are still attached to the cell surface. The supernatant was removed and cells were extensively washed, followed by further incubation with DMEM containing 0.02% BSA, 1% P/S and 40 *μ*M or 100 *μ*M importazole, respectively, for different times. Cells were then washed with PBS and the cytosolic RNA was isolated using an adjusted protocol of the RNeasy Kit (QIAGEN, Hilden, Germany). The efficiency of separation of cell lysates into cytoplasmic and nuclear fraction is depicted in S15 Fig.

#### RT-qPCR

Reverse transcription was performed using the Uni12 primer (5’-AGCRAAAGCAGG-3’ [35]) in combination with the standard protocol of the SuperScript III RT-polymerase (life-technologies). Absolute RNA quantification was obtained with the LightCycler 480 Instrument II (Roche, Mannheim Germany). The PCR reaction was carried out as following: pre-incubation for 5 min at 95°C followed by 45 cycles consisting of 10 sec at 95°C (melting), 10 sec at 57°C (annealing) and 30 sec at 72°C (amplification). The PCR solution was prepared according to the SYBR Green Kit protocol (Peqlab, Erlangen Germany). For the quantification of the copy number of viral RNA we used a primer which specifically binds to wild type and mutant H3 HA (Fwd Primer: TGACCAAATCAGGAAGCACA, Rev Primer: GGAGCGATTAGGTTCCCATT) and a plasmid containing H3 HA as a reference.

#### Segment-specific PCR

RNA from 100 *μ*l IAV X31 produced in chicken eggs was isolated using the RNeasy Kit (QIAGEN, Hilden, Germany). Reverse transcription was applied as mentioned above using the Uni12 primer. For the segment specific PCR, following primer pairs were used (5’-3’): PB2-Fwd: AGCGAAAGCAGGTCAATTAT; PB2-Rev: AGTAGAAACAAGGTCGTTTTTAAAC; NP-Fwd: AGCAAAAGCAGGGTAGATAATC; NP-Rev: AGTAGAAACAAGGGTATTTTTC; HA-Fwd: AGCAAAAGCAGGGGAAAA; HA-Rev: AGTAGAAACAAGGGTGTTTTTC. 3 *μ*l cDNA was mixed with 4 *μ*l 5x HF Phusion-Buffer, 1 *μ*l dNTPs (10mM), 0.4 *μ*l MgCl2 (50mM), 0.5 *μ*l Fwd and Rev primer (20 *μ*M), 3 *μ*l Phusion high fidelity DNA polymerase (Thermo Scientific, Darmstadt, Germany) and water was added to a total reaction volume of 20 *μ*l. PCR reaction mix was initially denaturated for 3 min at 98°C followed by 35 cycles starting with an additional denaturation step at 98°C for 25sec, 45sec at 60°C (55°C for HA) and 2 min at 72°C. The final elongation step was set at 72°C for 10 min (Primer and PCR conditions were modified from [18]). PCR products were visualized using gel electrophoresis.

### Computational methods

#### Model structure

For the non-spatial model of the virus entry, we used ordinary differential equations to describe the dynamics of the intracellular model species. We chose a level of detail that is as coarse as possible but can still be compared with experimental measurements.

The dynamics of the model species is determined by kinetic laws that govern the increase and decrease depending on rate constants and other model species. For most biochemical reactions, the law of mass action represents a good approximation even if the conditions of spatial homogeneity are not fulfilled. Thus, we modeled all transport reactions using this approach.

In order to reproduce the observed pH sensitivity of the endosome-virus fusion, however, a kinetic law expressing a switch-like behavior in the pH response was required. The formula for Hill kinetics [23], as known from enzyme kinetics, exhibits this requested behavior and was used in the corresponding equation. With this, the following set of ODEs could be deployed:

$$\begin{array}{lll} \frac{\text{d}[{\text{VirRec}}_{\text{ex}}]}{\text{d}t} & = & -k_{\text{end}}\cdot {\text{VirRec}}_{\text{ex}}\\ \frac{\text{d}[{\text{VirRec}}_{\text{end}}]}{\text{d}t}\; & = & k_{\text{end}}\cdot {\text{VirRec}}_{\text{ex}}-k_{\text{basal}}\cdot [{\text{VirRec}}_{\text{end}}]-k_{\text{fus}}\cdot \frac{{\left[{\text{H}}_{\text{end}}^{\text{+}}\right]}^{h}\cdot [{\text{VirRec}}_{\text{end}}]}{{\left[{\text{H}}_{\text{end}}^{\text{+}}\right]}^{h}+k_{\text{H+}}{}^{h}}\\ \frac{\text{d}[{\text{vRNP}}_{\text{cyt}}]}{\text{d}t} & = & k_{\text{basal}}\cdot [{\text{VirRec}}_{\text{end}}]+k_{\text{fus}}\cdot \frac{{\left[{\text{H}}_{\text{end}}^{\text{+}}\right]}^{h}\cdot [{\text{VirRec}}_{\text{end}}]}{{\left[{\text{H}}_{\text{end}}^{\text{+}}\right]}^{h}+k_{\text{H+}}{}^{h}}-k_{\tau }\cdot [{\text{vRNP}}_{\text{cyt}}]-k_{\text{deg}}\cdot [{\text{vRNP}}_{\text{cyt}}]\\ \frac{\text{d}[{\text{vRNP}}_{\text{cyt1}}]}{\text{d}t} & = & k_{\tau }\cdot [{\text{vRNP}}_{\text{cyt}}]-k_{\tau }\cdot [{\text{vRNP}}_{\text{cyt1}}]-k_{\text{deg}}\cdot [{\text{vRNP}}_{\text{cyt1}}]\\ \frac{\text{d}[{\text{vRNP}}_{\text{cyt2}}]}{\text{d}t}\; & = & k_{\tau }\cdot [{\text{vRNP}}_{\text{cyt1}}]-k_{\tau }\cdot [{\text{vRNP}}_{\text{cyt2}}]-k_{\text{deg}}\cdot [{\text{vRNP}}_{\text{cyt2}}]\\ \frac{\text{d}[{\text{vRNP}}_{\text{cyt3}}]}{\text{d}t} & = & k_{\tau }\cdot [{\text{vRNP}}_{\text{cyt2}}]-k_{\tau }\cdot [{\text{vRNP}}_{\text{cyt3}}]-k_{\text{deg}}\cdot [{\text{vRNP}}_{\text{cyt3}}]\\ \frac{\text{d}[{\text{vRNP}}_{\text{cyt4}}]}{\text{d}t} & = & k_{\tau }\cdot [{\text{vRNP}}_{\text{cyt3}}]-k_{\tau }\cdot [{\text{vRNP}}_{\text{cyt4}}]-k_{\text{deg}}\cdot [{\text{vRNP}}_{\text{cyt4}}]\\ \frac{\text{d}[{\text{vRNP}}_{\text{cyt5}}]}{\text{d}t} & = & k_{\tau }\cdot [{\text{vRNP}}_{\text{cyt4}}]-k_{\text{deg}}\cdot [{\text{vRNP}}_{\text{cyt5}}]-k_{\text{imp}}\cdot \frac{\left[{\text{vRNP}}_{\text{cyt5}}\right]}{k_{\text{inhib}}+1}\\ \frac{\text{d}[{\text{vRNP}}_{\text{nuc}}]}{\text{d}t} & = & k_{\text{imp}}\cdot \frac{\left[{\text{vRNP}}_{\text{cyt5}}\right]}{k_{\text{inhib}}+1} \end{array}$$

where [X] denotes the concentration of a model species X.

The dynamics of endosome acidification are expressed as *H*^+^ concentration:

$$\left[H_{\text{end}}^{+}\right]≔-\log_{10}\left(\left[{\text{pH}}_{\text{end}}\right]\right).$$

This concentration is increasing proportionally to the ATPase activity (*k*_ATPase_):

$$H_{\text{end}}^{+}=10^{-({\text{pH}}_{\text{lb}}+\left({\text{pH}}_{\text{ub}}-{\text{pH}}_{\text{lb}}\right)\exp\left(-k_{\text{ATPase}}\cdot t\right))}$$

where pH_ub_ and pH_lb_ and are the upper (directly after endocytosis) and lower bounds (matured endosome) of the pH range in the endosome.

The delay *τ* caused by diffusion is implemented in the ODE model using a linear chain of *N* = 5 reactions (model species [vRNP_cyt1–5_]) with simple mass action kinetics with the parameter

$$k_{\tau }=\frac{N}{\tau }$$

specifying the delay times [38].

The spatial model is structured according to the biological properties of an idealized MDCK cell as depicted in S9A Fig. The cell shape has been approximated by a cuboid with a longitudinal extent of 15 *μ*m and a lateral extent of 20 *μ*m and 30 *μ*m respectively. The nucleus of the host cell is represented by a sphere of 10 *μ*m in diameter that is located in lower half of the cuboid (center of the sphere at 9.5 *μ*m from the top).

Molecular crowding can affect diffusion of particles within mammalian cells. However, in many situations, diffusion of macro-molecules is still normal and isotropic but with the only difference that the effective speed is slower [39]. Therefore, we can use an effective diffusion coefficient for our spatial model as it was measured by single particle tracking of individual vRNPs in living cells [6].

The diffusion coefficient of vRNP complexes could be approximated from their 3D structure [40] using the Stokes-Einstein equation (Eq 2).

$$D=\frac{k_{B}T}{6\pi \;\eta \;r}$$ (2)

$$D_{\text{comp}}\approx 0.7\cdot D_{\text{vRNP}}$$ (3)

Using the value from [6], we obtain

$$D_{\text{vRNP}}=7.0\times 10^{-13}\;\frac{m^{2}}{s}$$ (4)

$$\Rightarrow D_{\text{comp}}\approx 4.9\times 10^{-13}\;\frac{m^{2}}{s}$$ (5)

#### ODE-Simulation and parameter estimation

To simulate the system we utilized the D2D-Toolbox [41] for MATLAB which also provides methods for parameter estimation from time course data. This toolbox is making use of the non-linear ODE solver CVODES of the SUNDIALS suite [42], which integrates the ODE system and its sensitivity equations simultaneously.

For parameter estimation, a deterministic optimization strategy (algorithm lsqnonlin in MATLAB) was applied to minimize the negative log-likelihood (LL)

$$-2\text{LL}=N\log\left(2\pi \right)+\sum_{i=1}^{N}\left[2\log\left(\sigma_{i}\right)+{\left(\frac{y_{i}-g\left(\vec{x},\vec{u},\theta ,t_{i}\right)}{\sigma_{i}}\right)}^{2}\right]$$

where *N* denotes the number of data points *y*_*i*_ with the corresponding variance $\sigma_{i}^{2}$. The model output *g* depends on the variables $\vec{x}$, inputs $\vec{u}$ and the parameters *θ*. For *σ* we use a parametric error model *σ*_*i*_ = *f*_*σ*_(*θ*, *t*_*i*_) that was defined depending on the measurement technique (e.g. proportional or constant error model).

To avoid convergence into local minima of the of the likelihood, initial parameters were sampled over several orders of magnitude using Latin hypercube sampling as described in [43]. For more details on the optimization methods please refer to [43] and [41]. A list of all kinetic model parameters and their optimal values as well as their confidence intervals is given in Table 1.

#### Spatial simulations

For stochastic simulation of the vRNP diffusion we used the STEPS [45] software package. The spatial simulation in this software is based on spatial extension of Gillespie’s SSA algorithm [46, 47] and is able to simulate reaction-diffusion-systems in a three-dimensional tetrahedral grid. We generated a geometry using standard 3D modeling software and performed a discretization of the volume using the tetgen mesh generator [48]. To determine the optimal sub-volume size of the tetrahedrons in the mesh we calculated the possible boundaries for sub-volume size and chose a maximal size of 0.9 fl out of this interval.

## Supporting Information

S1 Table Estimated parameter values. (PDF)

S1 Text Comparison of analytic and numeric solutions. (PDF)

S1 Fig Intracellular virus-endosome fusion as observed by dequenching of membrane-incorporated R18. MDCK cells were incubated with R18-labeled influenza virus for 10 min at 4°C, washed and R18 was detected using a confocal fluorescence microscopy. Surface plots of summed z-stacks were then constructed using ImageJ. A clear increase of fluorescence after 20 min can be observed in the case of the untreated control. In contrast, no dequenching was detectable after pre-incubation with 200nM bafilomycin A.
(TIF)

S2 Fig Quantification of spontaneous probe exchange between R18-labeled virus and endosomal membrane. MDCK cells were incubated with R18 labeled virus for 10 min at 4°C, washed and R18 was detected using confocal fluorescence microscopy. Viral fusion was inhibited by pre-incubating the cells in 200nM bafilomycin for 2h. The drug was present for the duration of the experiment.
(TIF)

S3 Fig pH dependency of IAV X-31 and WSN H3 wt virus-cell fusion. R18-labeled viruses were bound to human erythrocyte ghosts. Virus-cell fusion was measured by R18 fluorescence dequenching (FDQ) after pH lowering to the designated value. FDQ was normalized to the maximum value as described in Eq 1.
(TIF)

S4 Fig Identifiability analysis of the ODE model using likelihood profiles [44]. The parabola shaped parameter profiles are identifiable. The intersection of the profile (solid line) with the confidence threshold (dashed line) provides the confidence interval.
(TIF)

S5 Fig Simulated trajectories of the ODE model. The simulation was performed with an initial amount of one virus-receptor complex on the cell surface (see panel *VirRec_ex*). The gray lines in panel *VRNP_cyt1-5* correspond to the states forming the linear chain to account for the delay through diffusion. The amount of vRNP complexes in the nucleus (panel *VRNP_nuc*) saturates at about 0.5.
(TIF)

S6 Fig Replication kinetics of WSN H3 wt and WSN H3 mut. Infection efficiency as measured by viral NP accumulation 5h post-infection (A) (compare Fig 3) and growth curves performed over a duration of 72h (B) clearly indicate attenuated growth of WSN H3 mut. The HA titers of both utilized virus samples were identical (C).
(TIF)

S7 Fig Spatial progression of intracellular influenza A virus-endosome fusion events with respect to the nucleus. MDCK cells were incubated with R18-labeled influenza A X-31 virus for 10 min at 4°C, washed and R18 was detected using confocal fluorescence microscopy. a and c show overview images at 0 and 20 min post-infection. b and d show a zoomed representation of the highlighted areas in a and c. Nuclei are marked by a dashed line. Scale bars are (a, c) and 20 *μ*m (b, d) 5 *μ*m. Fusion events can be detected from R18 dequenching and their shortest distance to the nucleus was measured using ImageJ (e).
(TIF)

S8 Fig Position of the nucleus in MDCK cells under experimental conditions. MDCK cells were infected with influenza A/X31 virus at MOI 1 for 5h. The cells were fixed and immunolabeled against the viral nucleoprotein (NP). The nucleus was counterstained with DAPI. The recorded confocal stacks were projected for each channel and analyzed using CellProfiler (http://cellprofiler.org/). First, the nucleus was segmented using the DAPI image. Second, the cytoplasm was segmented with the NP image using Otsu thresholding. The outlines show good agreement with the cell borders visible in the bright field image.
(TIF)

S9 Fig Geometry of the spatial model is shaped after different cells modeled in a three-dimensional tetrahedral grid. (A) All results shown are calculated for a geometry that is supposed to resemble a typical MDCK cell with the given dimensions. (B) Additionally we modeled a lung epithelial cell. We generated a geometry using standard 3D modeling software and performed a discretization of the volume using the tetgen mesh generator [48]. To determine the optimal sub-volume size of the tetrahedrons in the mesh we calculated the possible boundaries for sub-volume size and chose a maximal size of 0.9 femtoliter(fl) for MDCK and 1.5fl for the lung epithelial cell out of this interval.
(TIF)

S10 Fig Parameter scans of the spatial model. For each of the tiles in the plots we simulated 1000 times with the respective parameters on the axis, the color gives the percentage of simulation in which at least one complete genome reached the nucleus. (A) Higher MOIs lead to more complete genomes in the nucleus. In the estimated range of degradation (blue line) we see a low percentage of complete genomes for lower MOIs (B) Distance and degradation have opposite effects on the number of genomes in the nucleus. With the estimated degradation we see a considerable effect of distance on the infection efficiency.
(TIF)

S11 Fig Experimental data (symbols) and resulting ODE model fits (solid lines) for degradation control experiment. The amount cytosolic vRNA was measured by quantitative RT-PCR after infection as a control for the nuclear import inhibition experiment (Fig 2, lower left panels). The shaded areas represent the estimated experimental error based on a parametric error model (see Materials and Methods). All y-axes show signal intensities in arbitrary units (a.u.).
(TIF)

S12 Fig Presence of viral full-length as well as defective RNA in influenza A/X31. A segment-specific PCR was performed for PB2, HA and NP. For all segments we could detect the full length RNA. For PB2, we found an additional smaller PCR product, indicating the presence defective interfering RNA. For HA/NP, for which RNA/protein accumulation was used to quantify virus-entry kinetics (results in Fig 2), we could not detect significant amplification of small PCR products.
(TIF)

S13 Fig Morphology of the cytoskeleton after cell treatment for the quantitative analysis of virus entry dynamics. MDCK cells were treated as during the virus entry experiments shown in Fig 2, fixed at the indicated conditions and stained for *α*-tubulin and actin. After both, a short incubation on ice (right column) as well as including a subsequent increase in temperature (center column), we could not detect an effect on the cytoskeleton (control, left column).
(TIF)

S14 Fig Effect of serum starvation and low-temperature incubation on endocytosis and endosome trafficking. MDCK cells were treated as indicated. Incubation in serum-free medium was performed for 30 min, incubation on ice for 20 min. The cells were washed and incubated with 0.5 mg/ml fluorescent dextran (Tetramethylrhodamine) for 20 min at 37°C to monitor total endocytosis and trafficking dynamics. Compared with control cells (left column), serum-starvation as well as low-temperature incubation had no detectable effect on number and position of internal vesicles.
(TIF)

S15 Fig Separation of cell lysates into cytoplasmic and nuclear fraction as verified by Western blot analysis. Cells were seeded in a 10cm dish one day prior to the experiment. Cytoplasmic lysis was performed by using an adjusted protocol of the RNeasy Kit (Qiagen). The cell lysate was centrifuged for 2 min at 300× g at 4°C and the supernatant was removed (cytoplasmic fraction). The pellet (nuclear fraction) was washed three times and lysed according to the standard RNeasy Kit protocol. From each fraction, 4 *μ*g protein were separated on a 10% SDS gel and blotted on a nitrocellulose membrane. Cytoplasmic (*α*-tubulin) and nuclear (fibrillarin) proteins in each fraction were detected using specific antibodies and fluorescence intensities were quantified using Image Studio (LICOR Biotechnology). The data plotted represent the mean of normalized fluorescence intensities of three independent experiments.
(TIF)

S16 Fig Comparison of the analytic and the numeric calculation of the mean first passage time (MFPT) of a particle within the cytoplasm. For one individual vRNP the MFPT is the smallest (left panel). MFPT for vRNP complexes (middle) is slower than for one individual vRNP but still faster than for a complete set of 8 vRNPs (right).
(TIF)
