# Supplementary material for: Viral RNA Degradation and Diffusion Act as a Bottleneck for the Influenza A Virus Infection Efficiency
Source: PLoS Comput Biol. 2016 Oct 25;12(10):e1005075. doi: 10.1371/journal.pcbi.1005075 (PMC5079570; doi:10.1371/journal.pcbi.1005075)
Supplement: S1 Table — (PDF) [file pcbi.1005075.s001.pdf]

index	name	θ_{lb}	$\hat{\theta}$	θ_{ub}	non-log $\hat{\theta}$
1	k_ATPase	-5	-1.1001	+3	$+7.94 \cdot 10^{-02}$
2	h	-5	+0.2796	+3	$+1.90 \cdot 10^{+00}$
3	h_WSN_H3_mut	-5	+0.6285	+3	$+4.25 \cdot 10^{+00}$
4	h_WSN_H3_wt	-5	+0.3599	+3	$+2.29 \cdot 10^{+00}$
5	init_Endosomes_FDQ	-5	-4.8812	+3	$+1.31 \cdot 10^{-05}$
6	init_VirRec_end_140807	-5	-2.1374	+3	$+7.29 \cdot 10^{-03}$
7	init_VirRec_end_140822	-5	-1.1345	+3	$+7.34 \cdot 10^{-02}$
8	init_VirRec_end_150604	-5	-0.5521	+3	$+2.80 \cdot 10^{-01}$
9	init_VirRec_end_150612	-5	-3.6796	+3	$+2.09 \cdot 10^{-04}$
10	init_Virus_number_X31	-5	+0.5787	+3	$+3.79 \cdot 10^{+00}$
11	init_Virus_number_X31_nuclear_NP	-5	+0.7553	+3	$+5.69 \cdot 10^{+00}$
12	init_Virus_number_X31_pH_end	-5	-4.3996	+3	$+3.98 \cdot 10^{-05}$
13	k_Hplus	-7	-5.3133	+3	$+4.86 \cdot 10^{-06}$
14	k_Hplus_WSN_H3_mut	-7	-5.6787	+3	$+2.10 \cdot 10^{-06}$
15	k_Hplus_WSN_H3_wt	-7	-5.3621	+3	$+4.34 \cdot 10^{-06}$
16	k_basal	-5	-2.0345	+3	$+9.24 \cdot 10^{-03}$
17	k_deg	-5	-0.7242	+3	$+1.89 \cdot 10^{-01}$
18	k_end	-5	-1.0307	+3	$+9.32 \cdot 10^{-02}$
19	k_fus	-5	-0.4449	+3	$+3.59 \cdot 10^{-01}$
20	k_imp	-5	+4.0000	+4	$+1.00 \cdot 10^{+04}$
21	k_inhib_100uM	-5	-3.7360	+3	$+1.84 \cdot 10^{-04}$
22	k_inhib_40uM	-5	+3.9953	+4	$+9.89 \cdot 10^{+03}$
23	k_tau	-5	+0.0821	+3	$+1.21 \cdot 10^{+00}$
24	offset_Fus_DiI_DiO	-5	-0.4321	+3	$+3.70 \cdot 10^{-01}$
25	offset_Fus_R18	-5	-1.2652	+3	$+5.43 \cdot 10^{-02}$
26	offset_NP	-5	-0.2488	+3	$+5.64 \cdot 10^{-01}$
27	offset_vRNP_140807	-5	-2.4147	+3	$+3.85 \cdot 10^{-03}$
28	offset_vRNP_140822	-5	-2.4716	+3	$+3.38 \cdot 10^{-03}$
29	offset_vRNP_150604	-5	-3.0353	+3	$+9.22 \cdot 10^{-04}$
30	offset_vRNP_150612	-5	-3.1259	+3	$+7.48 \cdot 10^{-04}$
31	pH_lb	-5	+0.6498	+3	$+4.46 \cdot 10^{+00}$
32	pH_ub	-5	+0.7926	+3	$+6.20 \cdot 10^{+00}$
33	scale_Fus_DiI_DiO	-5	-0.4557	+3	$+3.50 \cdot 10^{-01}$
34	scale_Fus_R18	-5	-0.3111	+3	$+4.89 \cdot 10^{-01}$
35	scale_NP	-5	-0.8445	+3	$+1.43 \cdot 10^{-01}$
36	scale_pH	-5	-0.7445	+3	$+1.80 \cdot 10^{-01}$
37	scale_vRNP_140807	-5	+0.0996	+3	$+1.26 \cdot 10^{+00}$
38	scale_vRNP_140822	-5	-0.8552	+3	$+1.40 \cdot 10^{-01}$
39	scale_vRNP_150604	-5	-2.7923	+3	$+1.61 \cdot 10^{-03}$
40	scale_vRNP_150612	-5	+1.0336	+3	$+1.08 \cdot 10^{+01}$
41	sd_FDQ_H3_mut	-5	-1.2285	+3	$+5.91 \cdot 10^{-02}$
42	sd_FDQ_H3_mut_rel	-5	-1.0824	+3	$+8.27 \cdot 10^{-02}$
43	sd_FDQ_WSN_H3_wt	-5	-1.1098	+3	$+7.77 \cdot 10^{-02}$
44	sd_FDQ_WSN_H3_wt_rel	-5	-1.4075	+3	$+3.91 \cdot 10^{-02}$
45	sd_FDQ_X31	-5	-1.8986	+3	$+1.26 \cdot 10^{-02}$
46	sd_FDQ_X31_rel	-5	-0.8759	+3	$+1.33 \cdot 10^{-01}$
47	sd_Fus_DiI_DiO	-5	-1.5136	+3	$+3.06 \cdot 10^{-02}$
48	sd_Fus_R18	-5	-1.2216	+3	$+6.00 \cdot 10^{-02}$
49	sd_NP	-5	-1.5560	+3	$+2.78 \cdot 10^{-02}$
50	sd_pH_MDCK	-5	-1.5457	+3	$+2.85 \cdot 10^{-02}$
51	sd_vRNP_140807	-5	-1.1650	+3	$+6.84 \cdot 10^{-02}$
52	sd_vRNP_140822	-5	-1.1602	+3	$+6.91 \cdot 10^{-02}$
53	sd_vRNP_150604	-5	-0.6037	+3	$+2.49 \cdot 10^{-01}$
54	sd_vRNP_150612	-5	-1.0648	+3	$+8.61 \cdot 10^{-02}$
55	sd_vRNP_tot_140807	-5	-1.6770	+3	$+2.10 \cdot 10^{-02}$
56	sd_vRNP_tot_140822	-5	-0.3950	+3	$+4.03 \cdot 10^{-01}$
57	sd_vRNP_tot_150604	-5	-0.9325	+3	$+1.17 \cdot 10^{-01}$
58	sd_vRNP_tot_150612	-5	-0.9742	+3	$+1.06 \cdot 10^{-01}$

S1 Table: Estimated parameter values. $\hat{\theta}$ indicates the estimated value of the parameters. θ_{lb} and θ_{ub} indicate the lower and upper bounds imposed during optimization. The non-log-column indicates the non-logarithmic value of the estimate. Parameters highlighted in red color indicate parameter values close to their bounds.
