# Supplementary material for: Viral RNA Degradation and Diffusion Act as a Bottleneck for the Influenza A Virus Infection Efficiency
Source: PLoS Comput Biol. 2016 Oct 25;12(10):e1005075. doi: 10.1371/journal.pcbi.1005075 (PMC5079570; doi:10.1371/journal.pcbi.1005075)
Supplement: S1 Text — (PDF) [file pcbi.1005075.s002.pdf]

S1 Text

Comparison of analytic and numeric solutions. In theory of stochastic processes, a quantity called *mean first passage time* (MFPT) can be calculated for a particle moving stochastically. In case of a one-dimensional geometry of length L with one reflecting and one absorbing boundary, the MFPT of a particle starting at position x at initial time $t = 0$ is given by

$$\tau(x) = \frac{L^2 - x^2}{2D} \tag{1}$$

where D denotes the diffusion coefficient of the particle [1].

Applied on our model geometry for a lung epithelial cell, the length $L = 35\,\mu\text{m}$ corresponds to the distance between the apical cell membrane and the top of the nucleus as shown in S9 Fig B.

However, for the case of the diffusion of 8 different vRNPs, the analytical solution for the MFPT is not available. Therefore, we simulated the one-dimensional scenario numerically as a random walk with a transition probability k that is proportional to the diffusion coefficient D and the time step τ

$$k = \sqrt{2d \cdot D\tau} \tag{2}$$

where d denotes the number of dimensions.

Analogously, we could record the time it takes for one particle to arrive at the nuclear membrane using our stochastic model in three dimensions with the cell-like geometry as described in the main text. The resulting distance-time relationship for both cases is depicted in S16 Fig. Here, we could see that for all initial distances larger zero, the MFPT was smaller in the one-dimensional calculation as a result of the simplified geometry. This explained the shift of the result of the 3D simulation (red lines) towards higher MFPT values.

The Brownian motion in 1D has been simulated in python. The 3D numeric simulation was performed using STEPS [2] with a simplified setup of the original reaction-diffusion model: dissociation of vRNP complexes has been disabled, degradation in the cytoplasm has been set to zero and the binding reaction of vRNPs to the nuclear membrane has been eliminated as well. The first passage time was then defined as the time that a particle is found for the first time close to the nuclear membrane (in one of the tetrahedrons adjacent to the nuclear membrane). For each initial distance, 1000 simulation runs have been performed. The MFPT was then calculated by taking the arithmetic mean over all runs.

[1] Gardiner C. Stochastic Methods: A Handbook for the Natural and Social Sciences. 4th ed. Berlin, Heidelberg: Springer; 2009.

[2] Hepburn I, Chen W, Wils S, De Schutter E. STEPS: efficient simulation of stochastic reaction-diffusion models in realistic morphologies. BMC systems biology. 2012 Jan;6(1):36.
