# Supplementary figures and images for: Viral RNA Degradation and Diffusion Act as a Bottleneck for the Influenza A Virus Infection Efficiency
Source: PLoS Comput Biol. 2016 Oct 25;12(10):e1005075. doi: 10.1371/journal.pcbi.1005075 (PMC5079570; doi:10.1371/journal.pcbi.1005075)

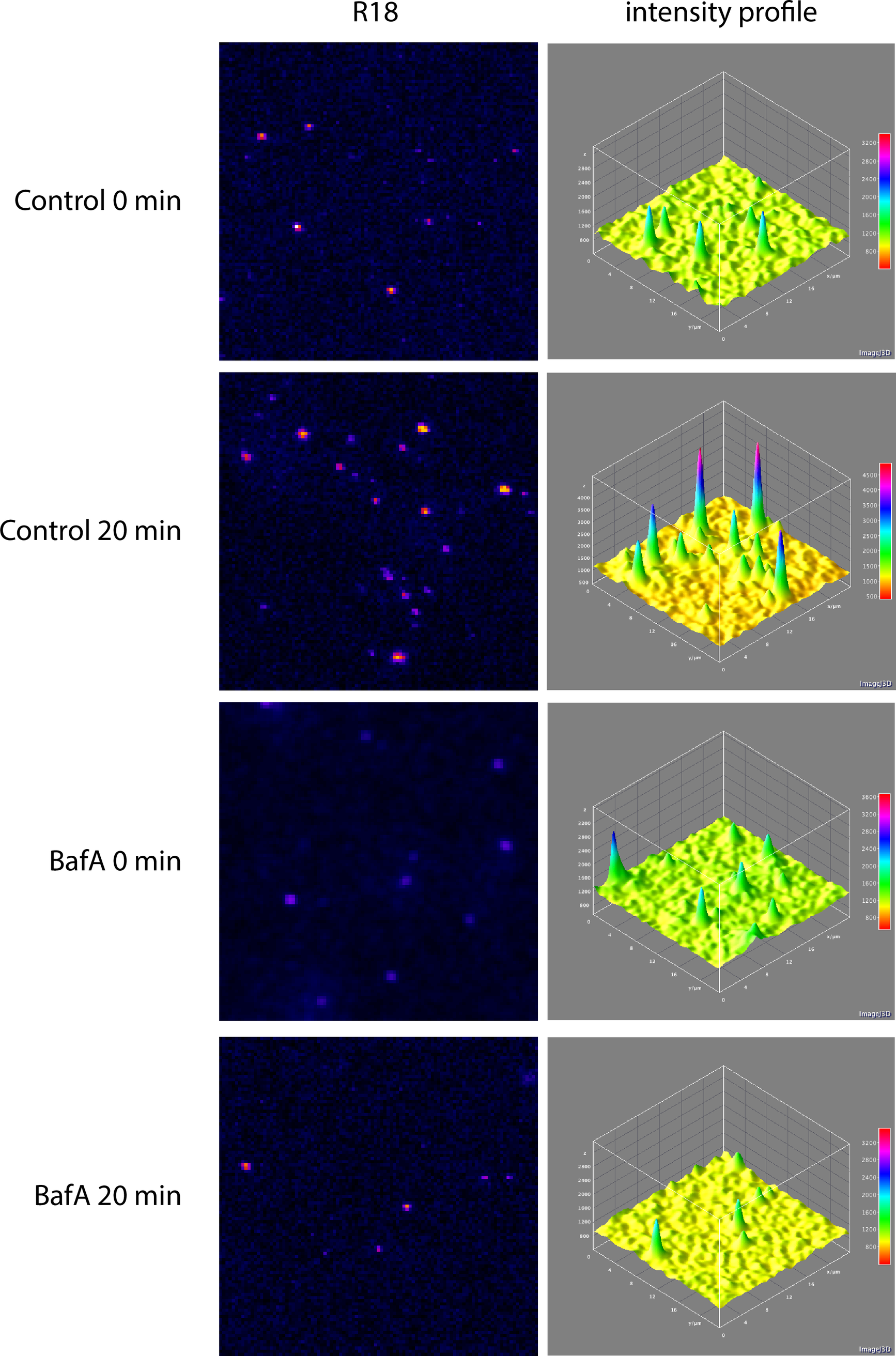

Supplement: S1 Fig — MDCK cells were incubated with R18-labeled influenza virus for 10 min at 4°C, washed and R18 was detected using a confocal fluorescence microscopy. Surface plots of summed z-stacks were then constructed using ImageJ. A clear increase of fluorescence after 20 min can be observed in the case of the untreated control. In contrast, no dequenching was detectable after pre-incubation with 200nM bafilomycin A. (TIF) [file pcbi.1005075.s003.tif]

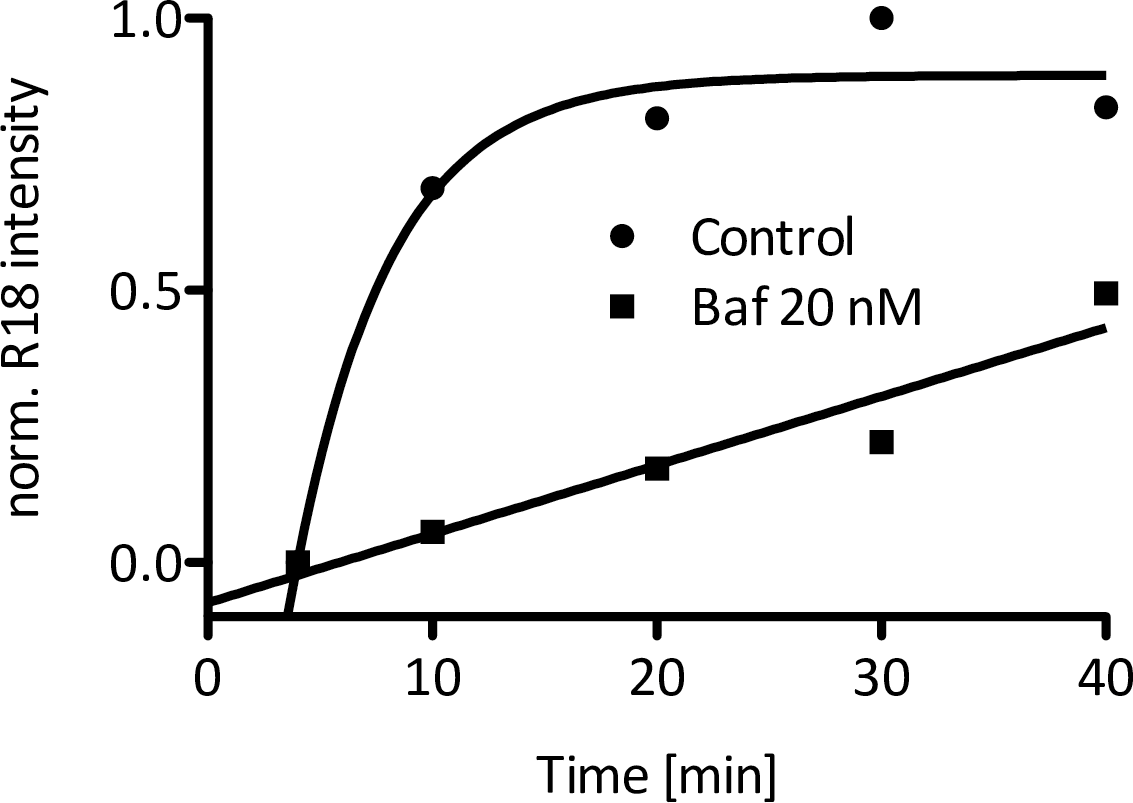

Supplement: S2 Fig — MDCK cells were incubated with R18 labeled virus for 10 min at 4°C, washed and R18 was detected using confocal fluorescence microscopy. Viral fusion was inhibited by pre-incubating the cells in 200nM bafilomycin for 2h. The drug was present for the duration of the experiment. (TIF) [file pcbi.1005075.s004.tif]

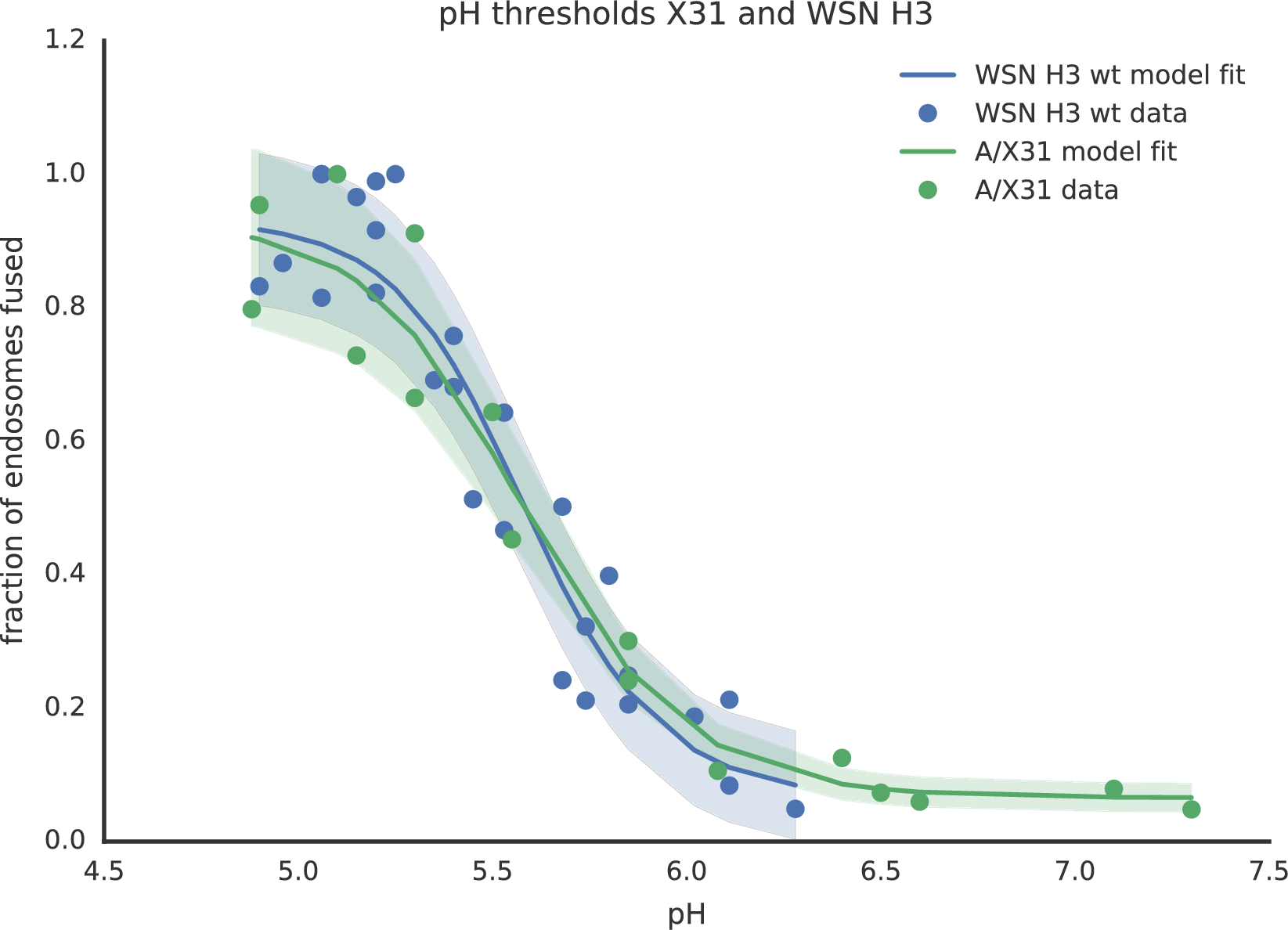

Supplement: S3 Fig — R18-labeled viruses were bound to human erythrocyte ghosts. Virus-cell fusion was measured by R18 fluorescence dequenching (FDQ) after pH lowering to the designated value. FDQ was normalized to the maximum value as described in Eq 1. (TIF) [file pcbi.1005075.s005.tif]

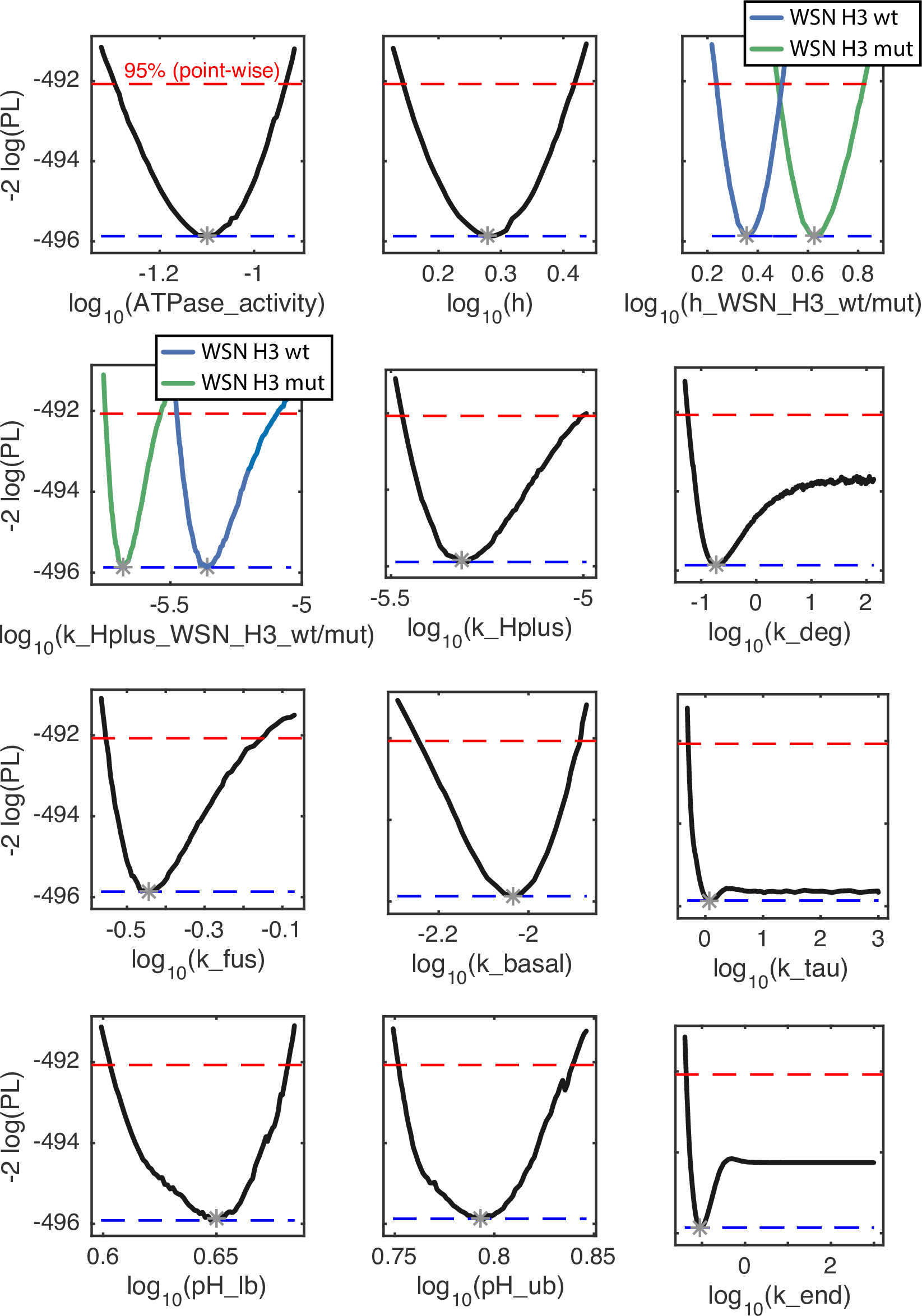

Supplement: S4 Fig — The parabola shaped parameter profiles are identifiable. The intersection of the profile (solid line) with the confidence threshold (dashed line) provides the confidence interval. (TIF) [file pcbi.1005075.s006.tif]

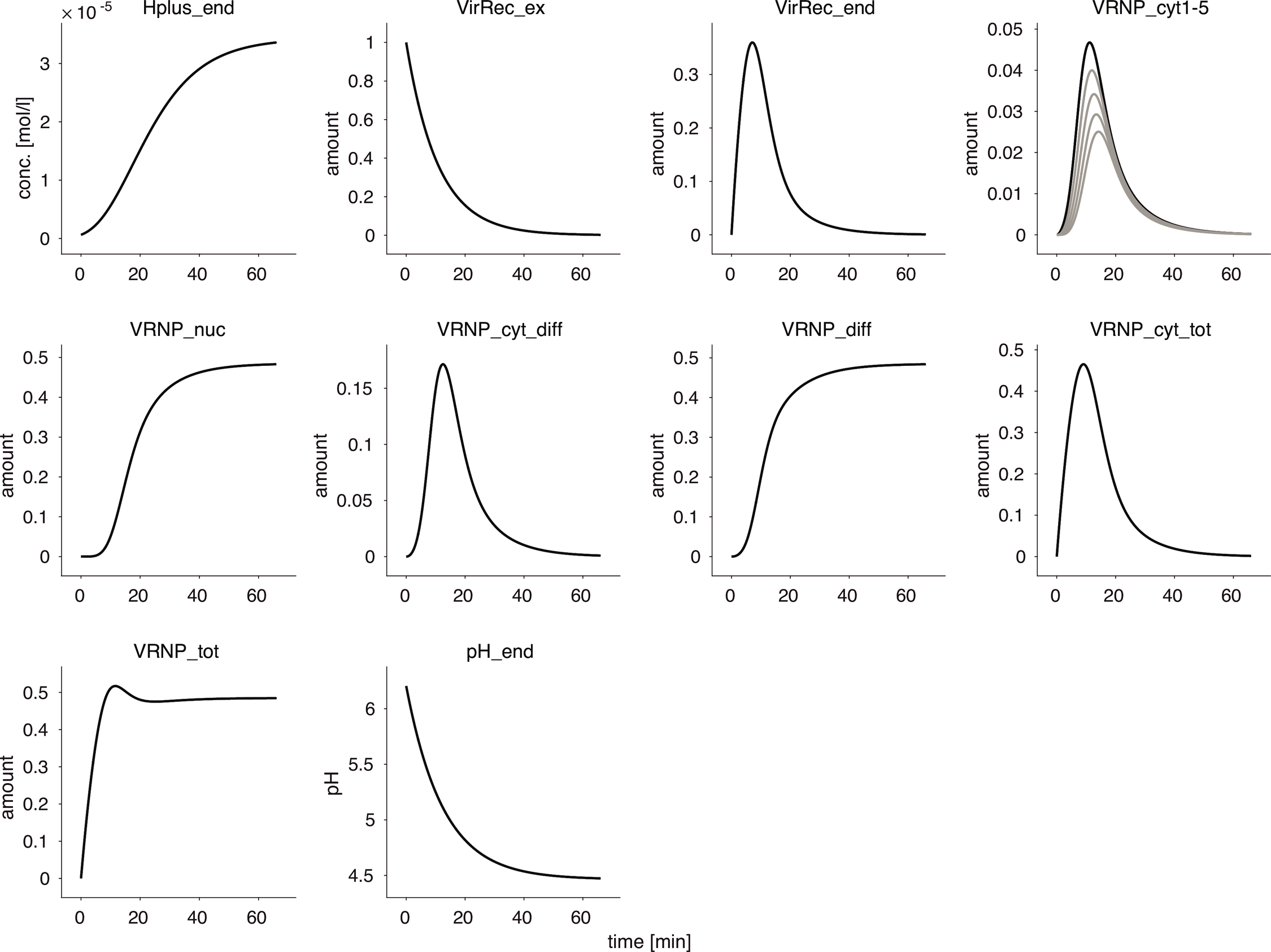

Supplement: S5 Fig — The simulation was performed with an initial amount of one virus-receptor complex on the cell surface (see panel VirRec_ex). The gray lines in panel VRNP_cyt1-5 correspond to the states forming the linear chain to account for the delay through diffusion. The amount of vRNP complexes in the nucleus (panel VRNP_nuc) saturates at about 0.5. (TIF) [file pcbi.1005075.s007.tif]

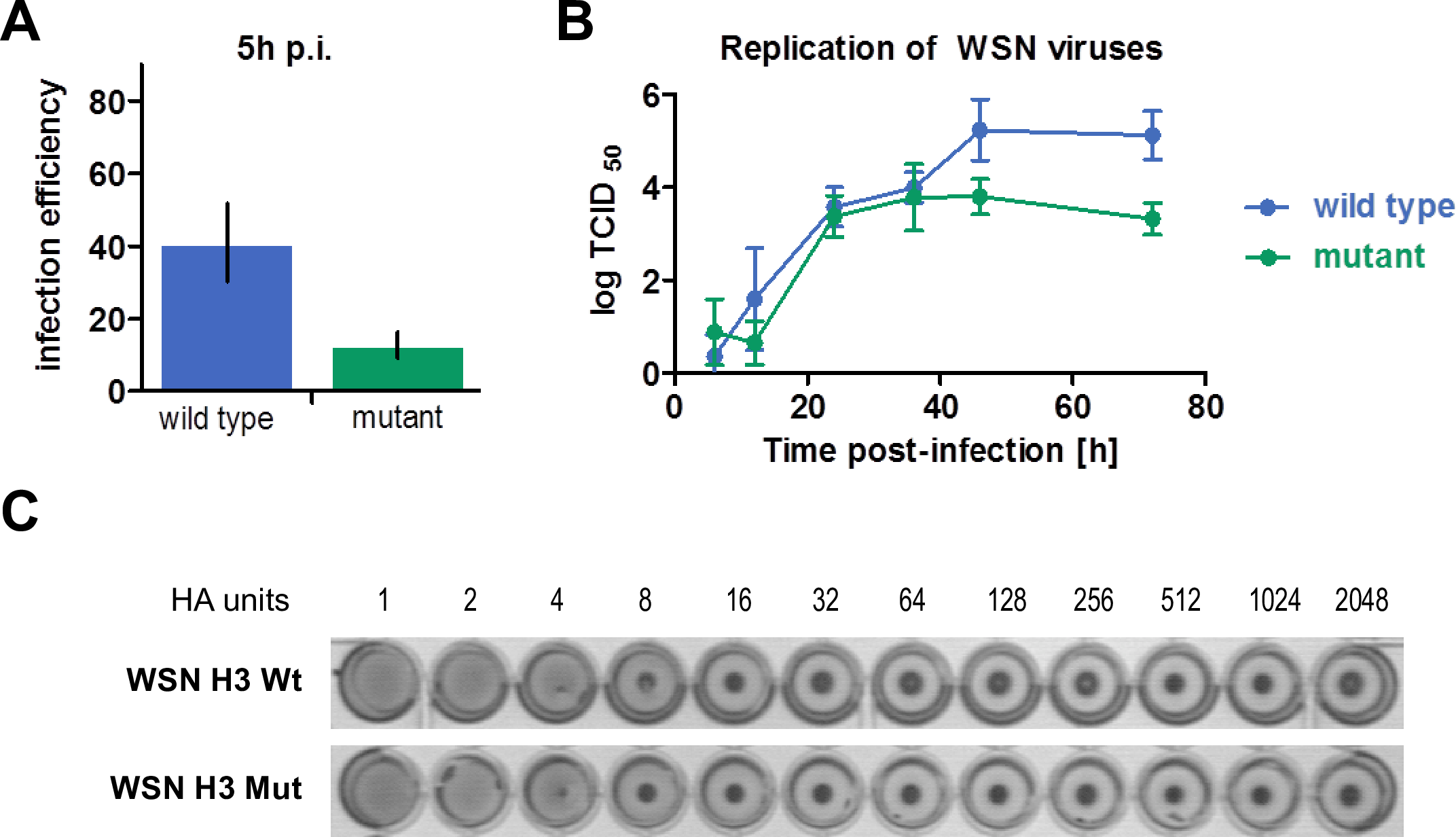

Supplement: S6 Fig — Infection efficiency as measured by viral NP accumulation 5h post-infection (A) (compare Fig 3) and growth curves performed over a duration of 72h (B) clearly indicate attenuated growth of WSN H3 mut. The HA titers of both utilized virus samples were identical (C). (TIF) [file pcbi.1005075.s008.tif]

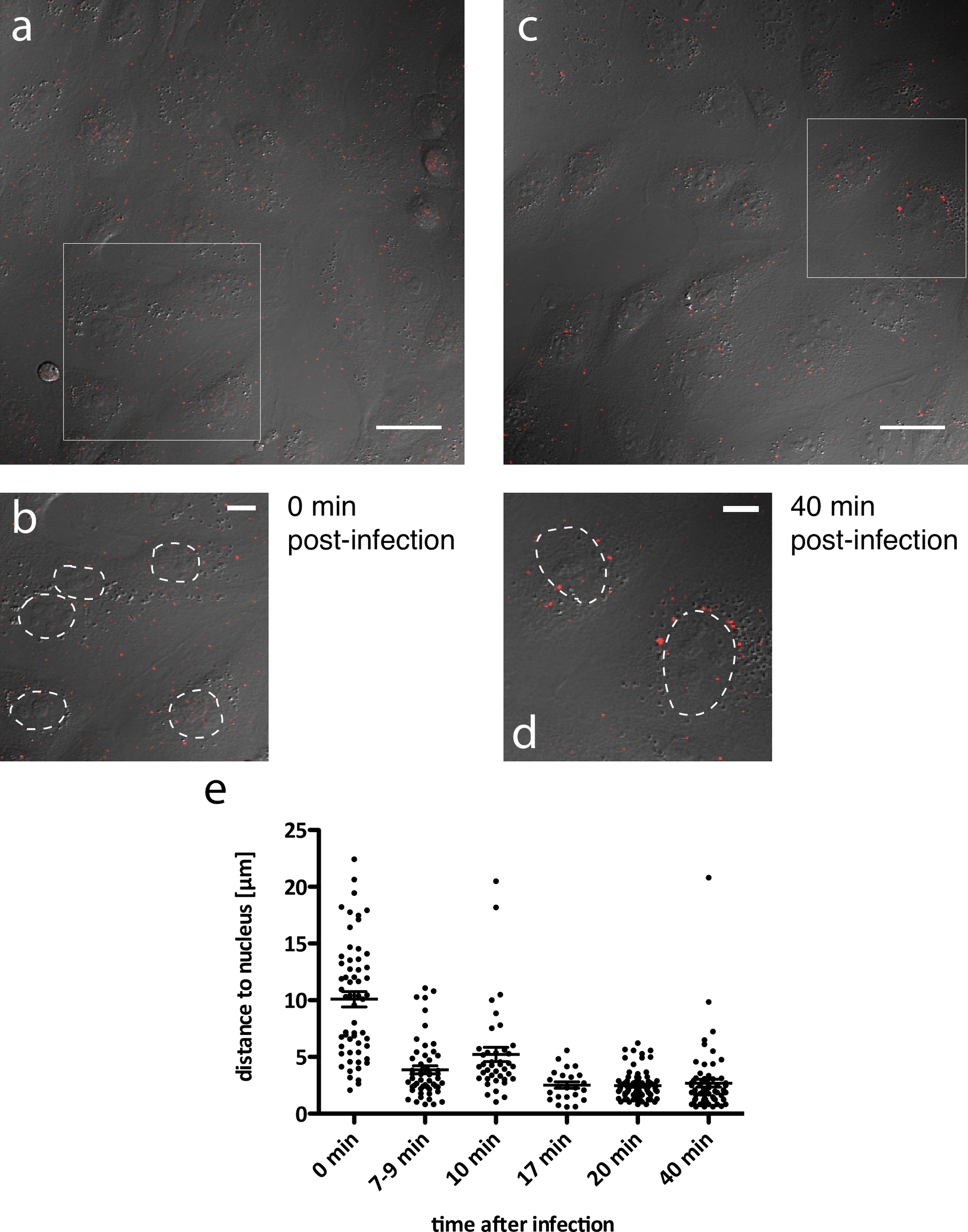

Supplement: S7 Fig — MDCK cells were incubated with R18-labeled influenza A X-31 virus for 10 min at 4°C, washed and R18 was detected using confocal fluorescence microscopy. a and c show overview images at 0 and 20 min post-infection. b and d show a zoomed representation of the highlighted areas in a and c. Nuclei are marked by a dashed line. Scale bars are (a, c) and 20 μm (b, d) 5 μm. Fusion events can be detected from R18 dequenching and their shortest distance to the nucleus was measured using ImageJ (e). (TIF) [file pcbi.1005075.s009.tif]

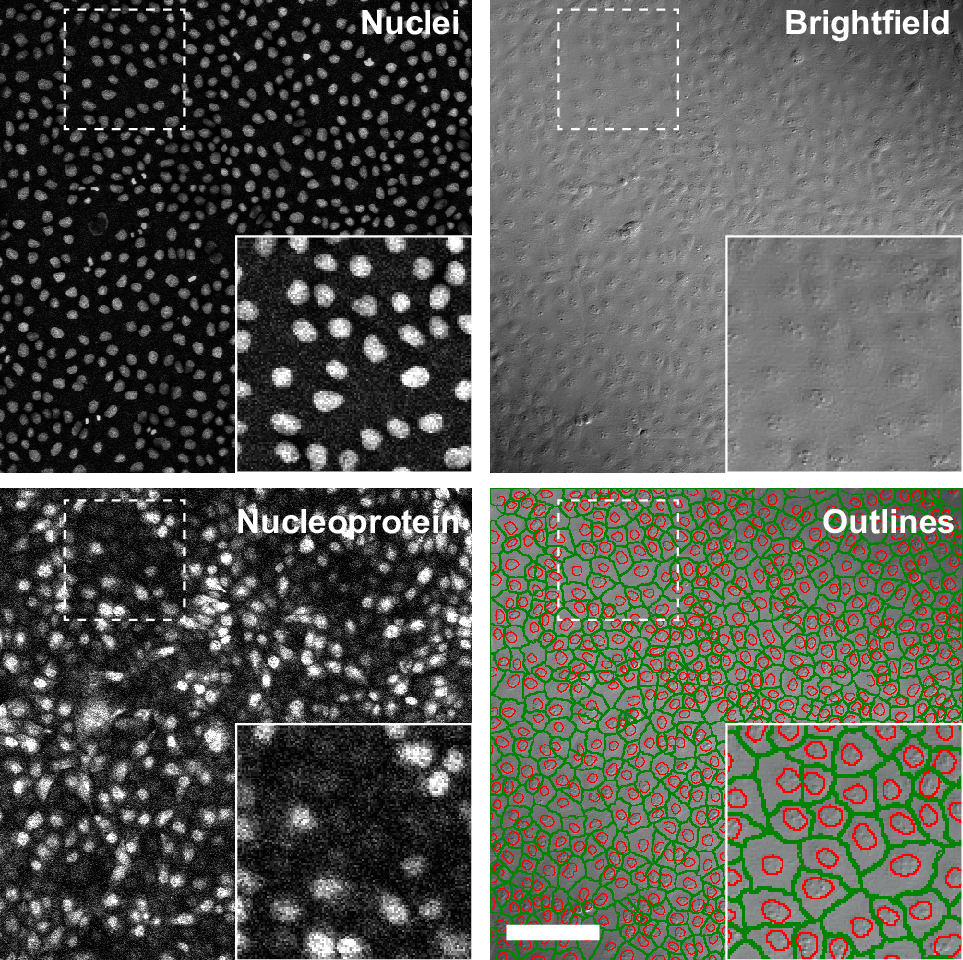

Supplement: S8 Fig — MDCK cells were infected with influenza A/X31 virus at MOI 1 for 5h. The cells were fixed and immunolabeled against the viral nucleoprotein (NP). The nucleus was counterstained with DAPI. The recorded confocal stacks were projected for each channel and analyzed using CellProfiler (http://cellprofiler.org/). First, the nucleus was segmented using the DAPI image. Second, the cytoplasm was segmented with the NP image using Otsu thresholding. The outlines show good agreement with the cell borders visible in the bright field image. (TIF) [file pcbi.1005075.s010.tif]

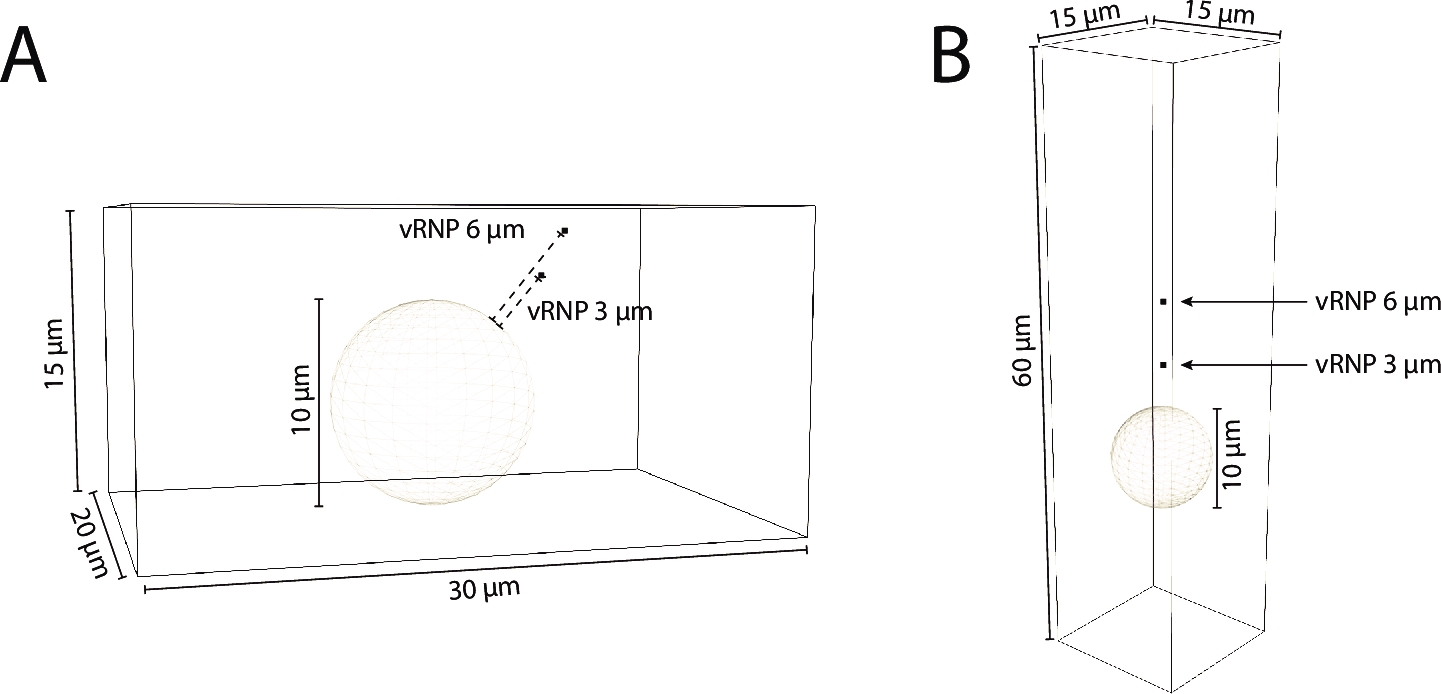

Supplement: S9 Fig — (A) All results shown are calculated for a geometry that is supposed to resemble a typical MDCK cell with the given dimensions. (B) Additionally we modeled a lung epithelial cell. We generated a geometry using standard 3D modeling software and performed a discretization of the volume using the tetgen mesh generator [48]. To determine the optimal sub-volume size of the tetrahedrons in the mesh we calculated the possible boundaries for sub-volume size and chose a maximal size of 0.9 femtoliter(fl) for MDCK and 1.5fl for the lung epithelial cell out of this interval. (TIF) [file pcbi.1005075.s011.tif]

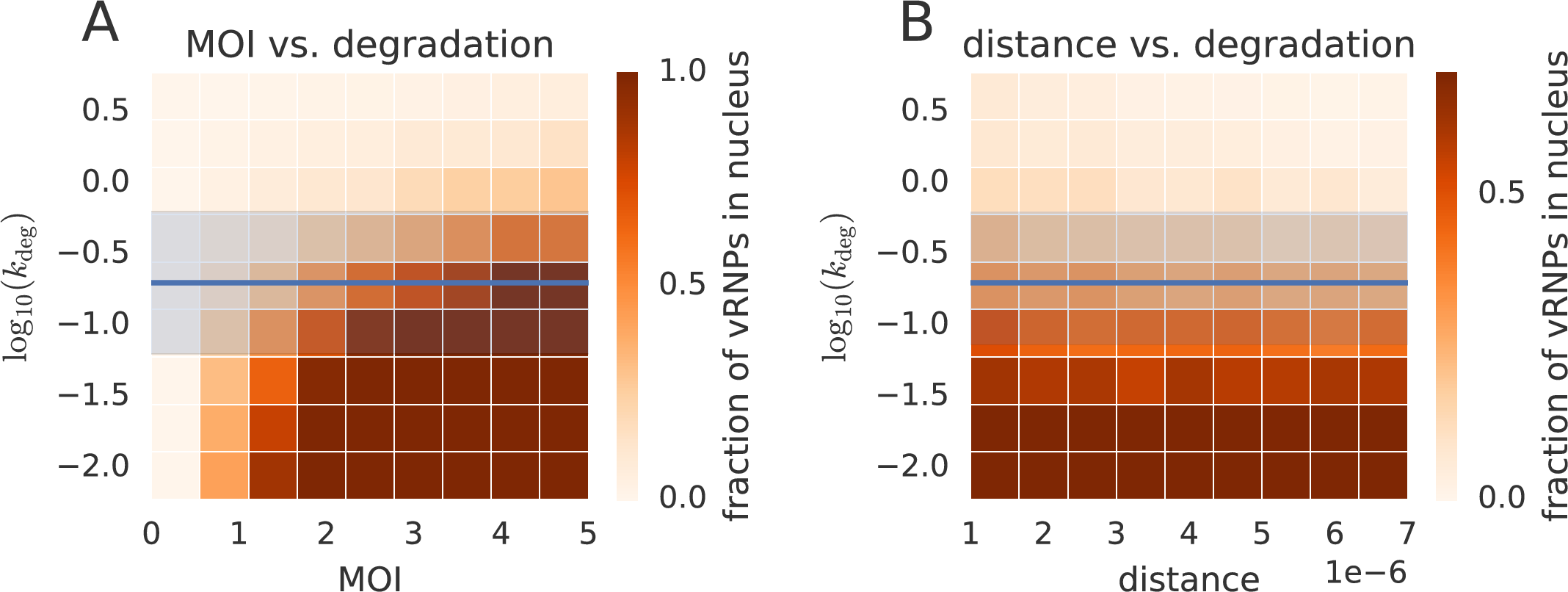

Supplement: S10 Fig — For each of the tiles in the plots we simulated 1000 times with the respective parameters on the axis, the color gives the percentage of simulation in which at least one complete genome reached the nucleus. (A) Higher MOIs lead to more complete genomes in the nucleus. In the estimated range of degradation (blue line) we see a low percentage of complete genomes for lower MOIs (B) Distance and degradation have opposite effects on the number of genomes in the nucleus. With the estimated degradation we see a considerable effect of distance on the infection efficiency. (TIF) [file pcbi.1005075.s012.tif]

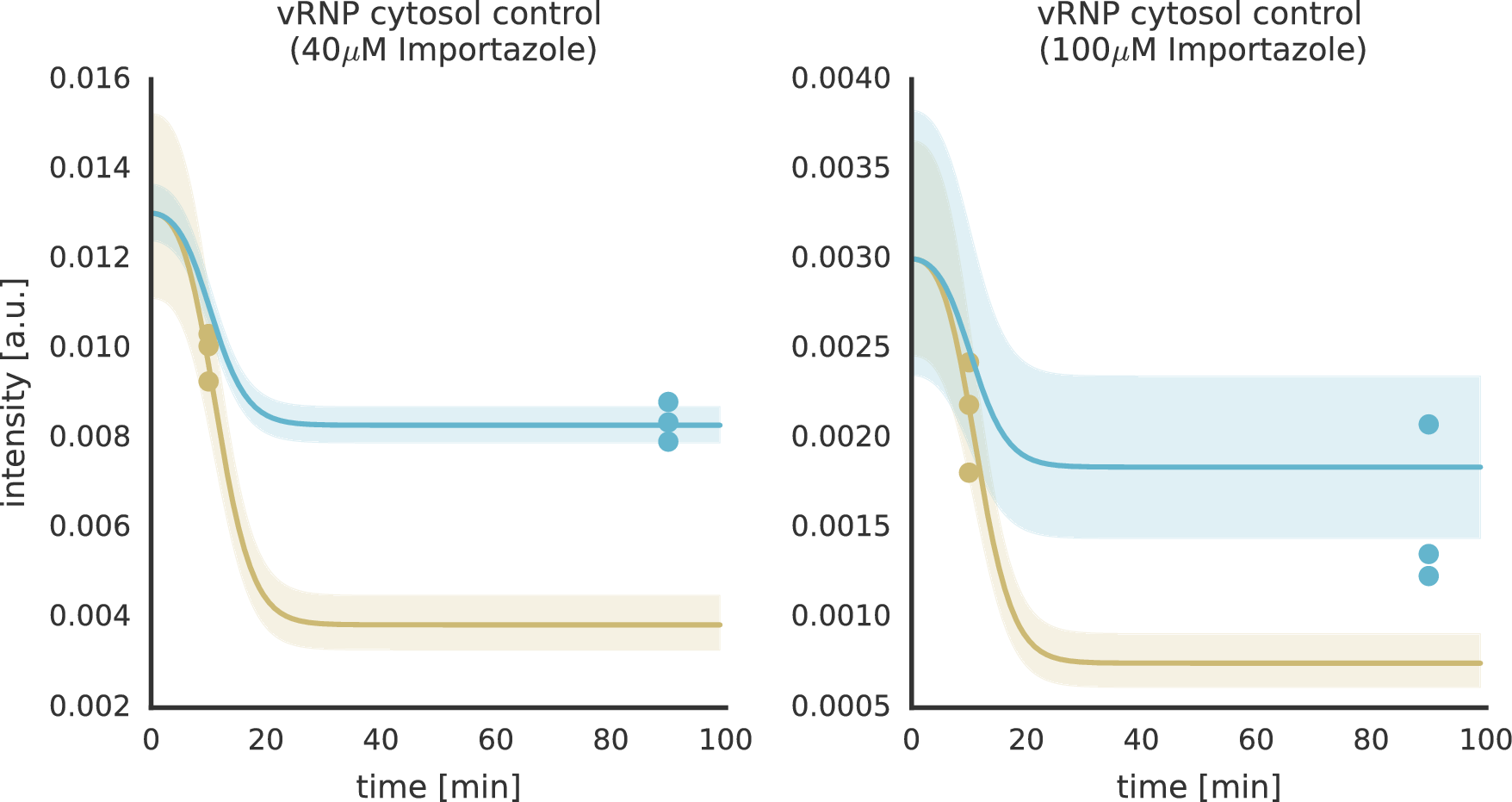

Supplement: S11 Fig — The amount cytosolic vRNA was measured by quantitative RT-PCR after infection as a control for the nuclear import inhibition experiment (Fig 2, lower left panels). The shaded areas represent the estimated experimental error based on a parametric error model (see Materials and Methods). All y-axes show signal intensities in arbitrary units (a.u.). (TIF) [file pcbi.1005075.s013.tif]

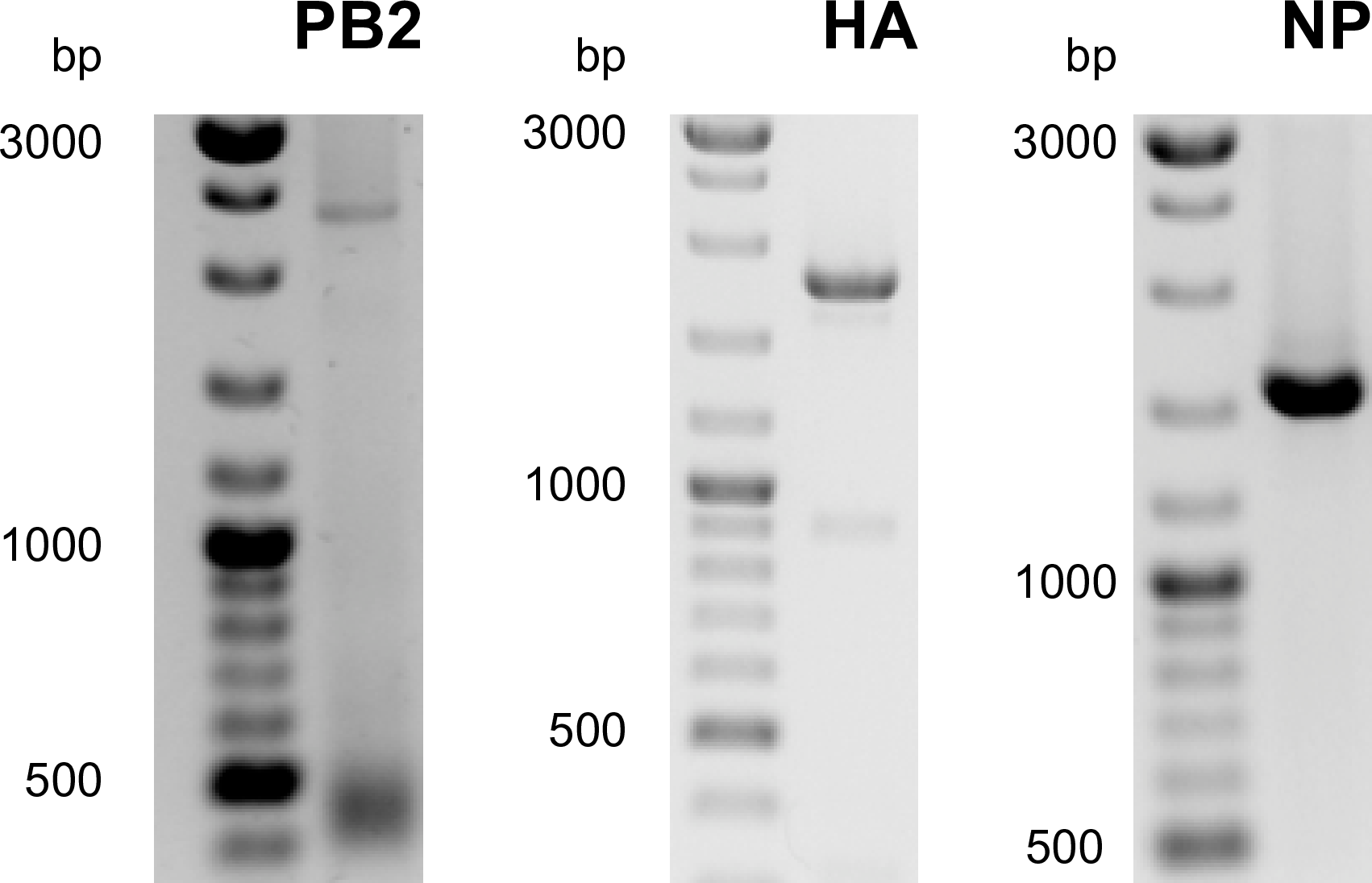

Supplement: S12 Fig — A segment-specific PCR was performed for PB2, HA and NP. For all segments we could detect the full length RNA. For PB2, we found an additional smaller PCR product, indicating the presence defective interfering RNA. For HA/NP, for which RNA/protein accumulation was used to quantify virus-entry kinetics (results in Fig 2), we could not detect significant amplification of small PCR products. (TIF) [file pcbi.1005075.s014.tif]

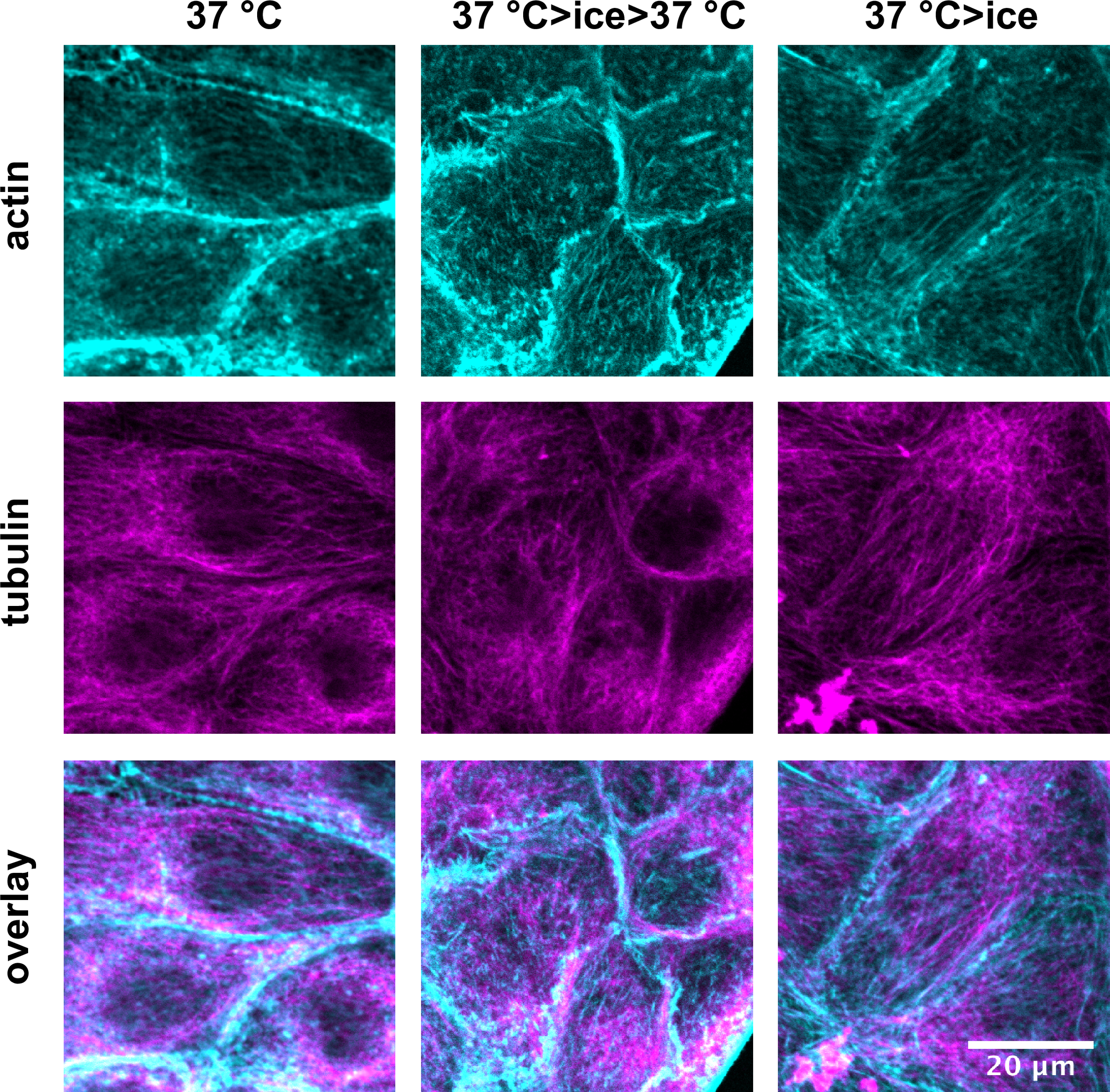

Supplement: S13 Fig — MDCK cells were treated as during the virus entry experiments shown in Fig 2, fixed at the indicated conditions and stained for α-tubulin and actin. After both, a short incubation on ice (right column) as well as including a subsequent increase in temperature (center column), we could not detect an effect on the cytoskeleton (control, left column). (TIF) [file pcbi.1005075.s015.tif]

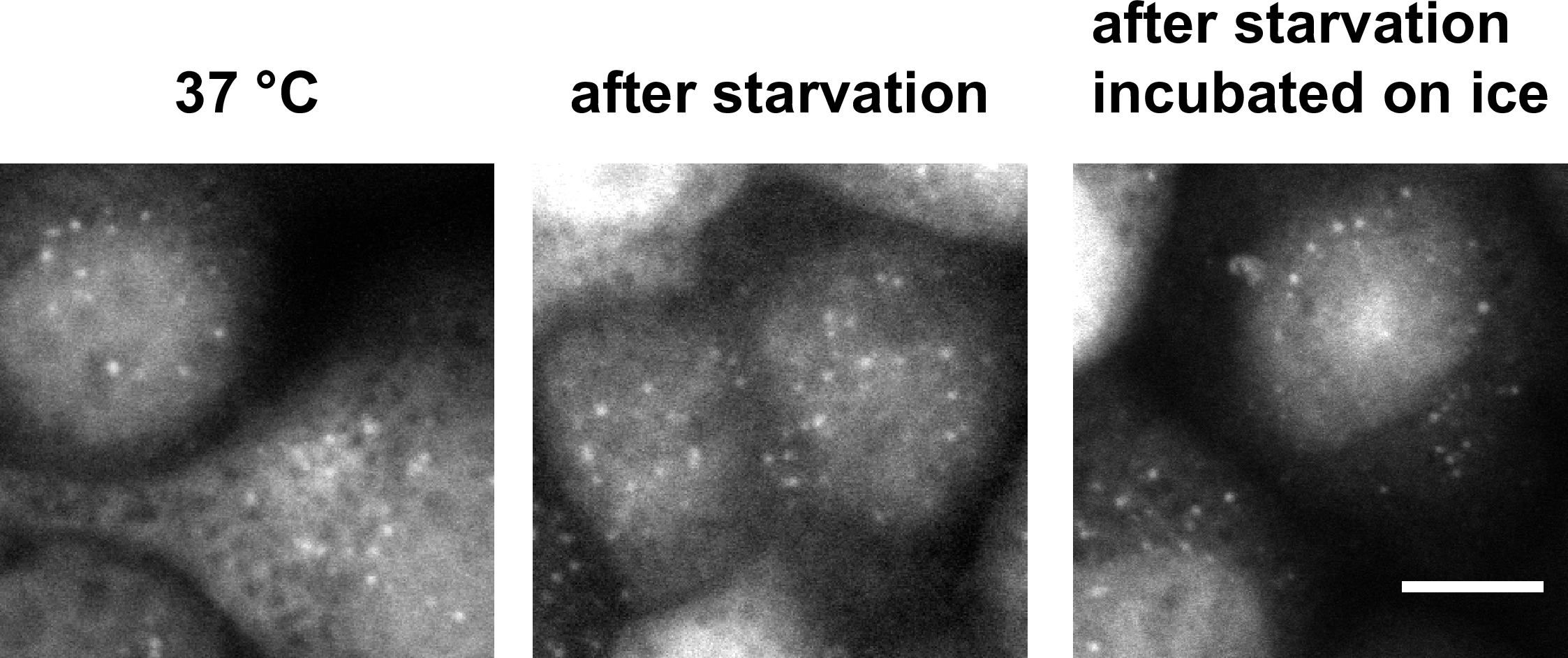

Supplement: S14 Fig — MDCK cells were treated as indicated. Incubation in serum-free medium was performed for 30 min, incubation on ice for 20 min. The cells were washed and incubated with 0.5 mg/ml fluorescent dextran (Tetramethylrhodamine) for 20 min at 37°C to monitor total endocytosis and trafficking dynamics. Compared with control cells (left column), serum-starvation as well as low-temperature incubation had no detectable effect on number and position of internal vesicles. (TIF) [file pcbi.1005075.s016.tif]

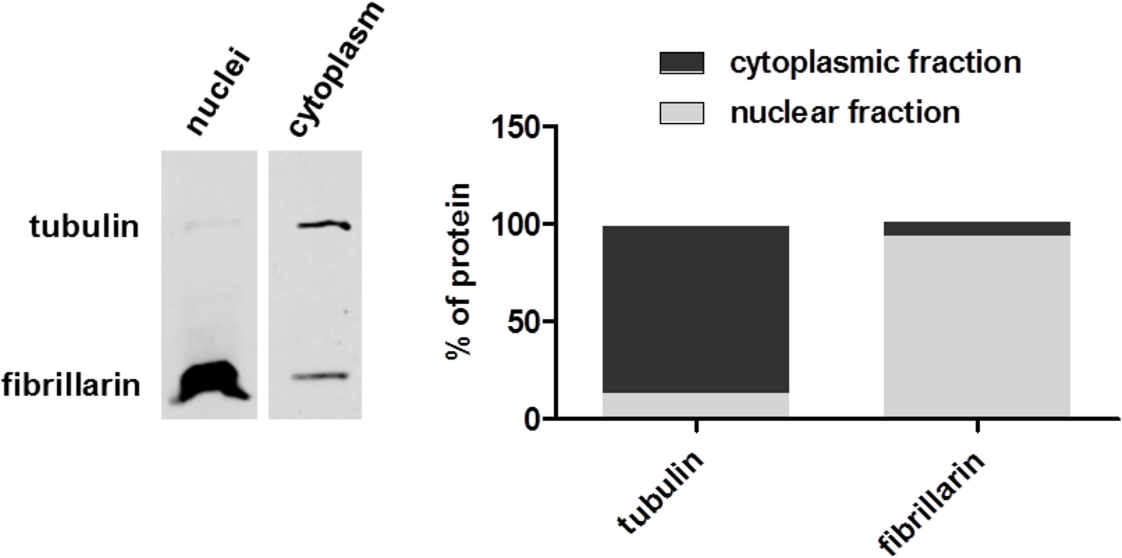

Supplement: S15 Fig — Cells were seeded in a 10cm dish one day prior to the experiment. Cytoplasmic lysis was performed by using an adjusted protocol of the RNeasy Kit (Qiagen). The cell lysate was centrifuged for 2 min at 300× g at 4°C and the supernatant was removed (cytoplasmic fraction). The pellet (nuclear fraction) was washed three times and lysed according to the standard RNeasy Kit protocol. From each fraction, 4 μg protein were separated on a 10% SDS gel and blotted on a nitrocellulose membrane. Cytoplasmic (α-tubulin) and nuclear (fibrillarin) proteins in each fraction were detected using specific antibodies and fluorescence intensities were quantified using Image Studio (LICOR Biotechnology). The data plotted represent the mean of normalized fluorescence intensities of three independent experiments. (TIF) [file pcbi.1005075.s017.tif]

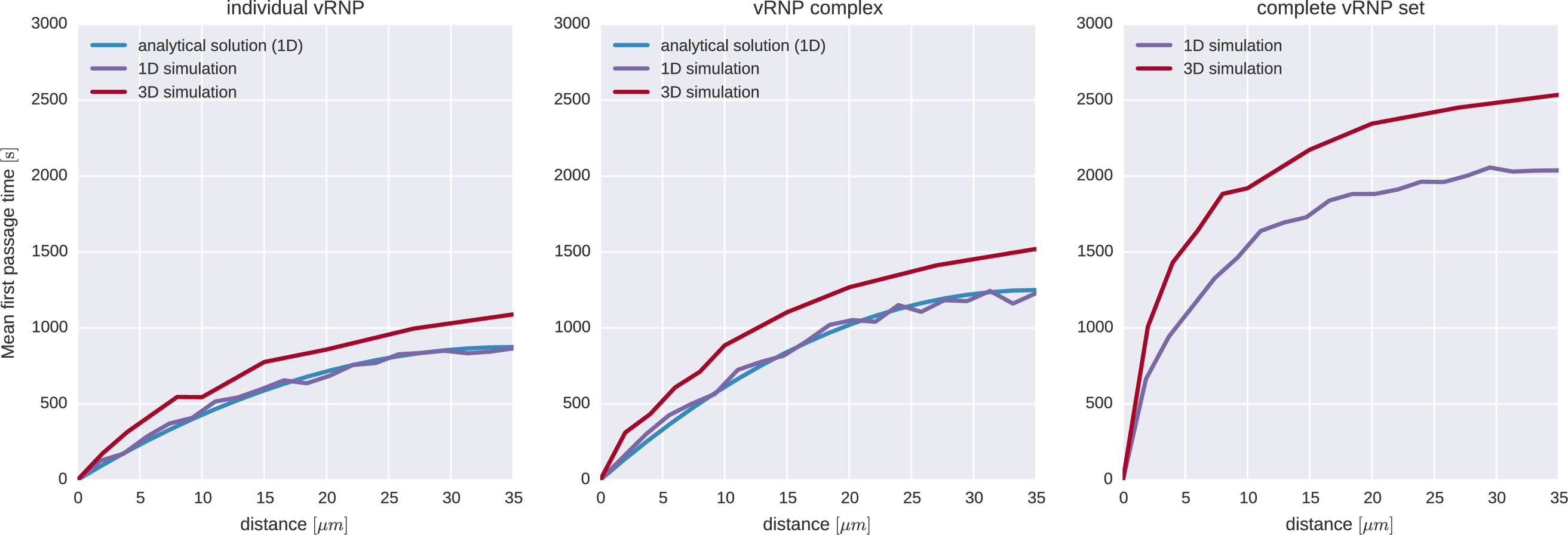

Supplement: S16 Fig — For one individual vRNP the MFPT is the smallest (left panel). MFPT for vRNP complexes (middle) is slower than for one individual vRNP but still faster than for a complete set of 8 vRNPs (right). (TIF) [file pcbi.1005075.s018.tif]
